# Structure-guided design of a bivalent SARS-CoV-2 mRNA vaccine with NTD stabilizing mutations enhances broad immunity

**DOI:** 10.3389/fimmu.2025.1718740

**Published:** 2026-01-22

**Authors:** Jinah Yeo, Mi-ran Yun, Seo-Yeon Kim, Jong-Hyun Seok, Ji Hyang Jeon, Taeyoung Lee, Jeonghun Kim, Kisoon Kim, Man-Seong Park, Dokeun Kim, You-Jin Kim

**Affiliations:** 1 Division of Infectious Disease Vaccine Research, Center for Vaccine Research, National Institute of Health, Korea Disease Control and Prevention Agency, Cheongju, Republic of Korea; 2 Department of Microbiology, College of Medicine, Korea University, Seoul, Republic of Korea; 3 Center for Vaccine Research, National Institute of Health, Korea Disease Control and Prevention Agency, Cheongju, Republic of Korea

**Keywords:** cross-variant immunity, mRNA vaccine, SARS-CoV-2, spike protein, stabilizing mutations, structure optimization

## Abstract

SARS-CoV-2 evolution, particularly the emergence of Omicron variants, has challenged vaccine efficacy, necessitating antigens with broad and variant-specific protection. To design mRNA vaccine antigens with broad-spectrum immunity and enhanced stability, we developed two spike antigens using *in silico* optimization: Css_dsg S, the ancestral strain–Delta variant consensus with stabilizing mutations, and Omi_dsg S, an Omicron-adapted design. Computational analysis identified two critical N-terminal domain stabilization sites consistently enhancing protein expression across variants, suggesting their potential as universal stabilizing elements. Css_dsg S elicited robust IFN-γ T cell responses and significantly elevated neutralizing antibody titers against variants in BALB/c mice. Omi_dsg S induced strong immune responses *in vivo*. A bivalent mRNA vaccine combining both antigens elicited superior neutralizing antibody responses and conferred enhanced protection against BN.1 and BA.5 challenges in K18-hACE2 mice. These findings support computationally optimized spike antigens, particularly the bivalent formulation, as a promising strategy for next-generation vaccines against SARS-CoV-2 variants.

## Introduction

Since its first emergence in Wuhan in December 2019, the severe acute respiratory syndrome coronavirus 2 (SARS-CoV-2) has caused more than 778 million cases of COVID-19 and more than 7 million deaths worldwide as of December 2025 (1). SARS-CoV-2 possesses a large RNA genome of approximately 30,000 nucleotides with a discontinuous nature of coronavirus transcription, resulting in high rates of recombination, insertions and deletions, and point mutations (2). These characteristics underlie the ongoing evolution of viruses and the emergence of multiple variants of concern (VOCs), including Alpha (B.1.1.7), Beta (B.1.351), Delta (B.1.617.2), and successive Omicron sublineages such as BA.1, BA.2, BA.4/5, XBB, and BQ.1.1 (3, 4). More recently, highly immune-evasive Omicron variants such as JN.1, KP.2, and FL.1.5.1 have become globally dominant, with JN.1, designated a WHO Variant of Interest (VOI) in December 2023, continues to circulate widely in 2024 and 2025 (5, 6). These emerging variants harbor numerous mutations in the spike (S) protein, particularly in the receptor-binding domain (RBD) and N-terminal domain (NTD), which increase transmissibility and allow evasion of neutralizing antibodies (7).

The RBD is the main target of neutralizing antibodies, exerting a significant effect on their activity; there are numerous studies on mutations in the RBD region. These mutations have significantly reduced the neutralizing capacity of the antibodies elicited by previous SARS-CoV-2 vaccinations (8–11), posing a substantial challenge to global public health efforts aimed at controlling the COVID-19 pandemic. However, accumulating evidence highlights the NTD as a critical antigenic region, which contributes to the formation of conformational epitopes and is recognized by a subset of potent neutralizing antibodies (9). Mutations in the NTD have been associated with altered antigenic properties and immune escape, highlighting the importance of targeting multiple spike regions for effective vaccine design. This has spurred research efforts to develop next-generation vaccines capable of inducing broad immune responses.

mRNA vaccine platforms, such as BNT162b2 (Pfizer/BioNTech) and mRNA-1273 (Moderna), which were initially developed against the ancestral Wuhan-Hu-1 strain, have demonstrated high efficacy in reducing severe disease and transmission rates (12–17). However, the efficacy of these first-generation vaccines is diminished against newly circulating variants that evade neutralizing antibodies because of mutations in the spike protein (18, 19). Although booster vaccines have been developed to match the spike protein sequences of circulating variants (20, 21), their effectiveness can vary as viral evolution continues. This highlights the need to develop vaccine antigens that aim to broaden neutralization breadth by leveraging conserved and structurally optimized features, thereby enhancing cross-variant protection.

A promising approach for achieving such broad protection is the rational design of spike antigens using computational methods. Recent advances in *in silico* vaccine design have enabled the prediction and optimization of antigen structures, allowing for the identification of mutations that enhance structural stability, antigenicity, and immunogenicity (22–25). These computationally guided strategies offer rapid and cost-effective means of developing optimized vaccine candidates by exploring the spike protein sequence space.

In this study, we applied a structure-guided computational approach to design spike antigens for mRNA vaccines targeting emerging SARS-CoV-2 variants, with the goal of enhancing cross-variant immune responses. Our strategy incorporated key stabilizing and variant-specific mutations to enhance cross-protection. By combining structural stabilization with targeted sequence optimization, we generated spike antigens with improved expression, stability, and immunogenicity. We further validated their immunogenicity and protective efficacy in mouse models, including K18-hACE2 transgenic mice challenged with Omicron variants. These findings highlight the potential of computationally guided spike antigen design as a strategy for developing next-generation SARS-CoV-2 vaccines with enhanced breadth of protection.

## Results

### *In silico* design of SARS-CoV-2 spike protein

**Figure 1:**
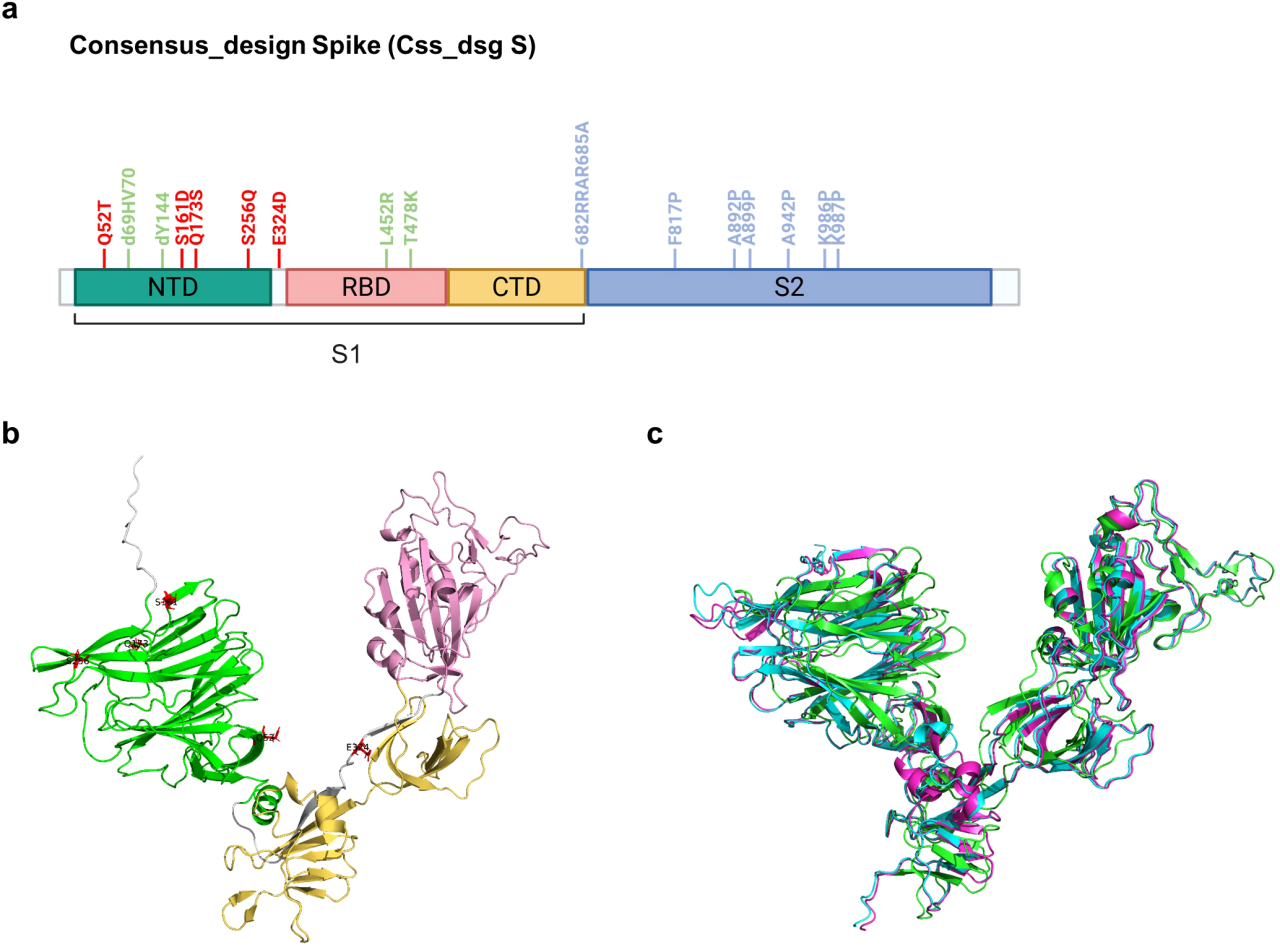
*In silico* design of SARS-CoV-2 spike protein. **(a)**, Features of variants (in light green), and substitution of cleavage site and known stabilized hexaPro (in skyblue) on consensus spike protein generated with sequence evolution analysis (created with BioRender.com). Design to increase structure stability (in red) on consensus spike protein. Residue positions are annotated based on the ancestral spike protein sequence. **(b)**, S1 subunit folded structure of consensus spike modeled using AlphaFold2, with indicated designed sites (induced mutations of Css_dsg S). **(c),** Superimposition of experimental (PDB: 6xr8), consensus, and *in silico* spikes. Experimentally solved 3D structure of spike (in green), consensus spike (in cyan), and *in silico* spike (in magenta) modeled using AlphaFold2.

In the present study, we used an *in silico* approach to design a broad-spectrum SARS-CoV-2 spike protein antigen with enhanced immunogenicity and cross-variant protection. The antigen design process integrates multiple computational strategies to optimize structural stability and functional properties. We applied a phylogenetically optimized mosaic antigen (POMA) approach (submitted) to generate a consensus sequence that incorporated conserved features across SARS-CoV-2 variants, spanning from the ancestral to Delta strain (light green in **Figure 1A**). The POMA approach is a phylogeny-based consensus design strategy that integrates conserved sequence features from representative SARS-CoV-2 variants to generate mosaic antigen sequences with broad coverage. To further enhance structural integrity, stabilizing mutations, including 6-P mutations in the S2 domain and cleavage site mutations, were introduced to maintain the pre-fusion conformation (sky blue in **Figure 1A**). Residue numbering for mutations was based on the ancestral reference spike sequence. This rationally designed antigen (consensus spike; Css S) is expected to provide broad immune response coverage across diverse variants.

To refine the structural stability of the spike protein, we used advanced structural modeling and designed an antigenic protein by introducing mutations. The S1 domain structure was predicted using AlphaFold2 and suboptimal stability regions were identified using pSUFER, allowing the identification of energetically favorable mutation sites primarily located within the NTD. For the residues identified by pSUFER, we utilized FuncLib, which generated a library of 1,000 spike protein designs with predicted improvements in structural stability. From this library, we selected five leading candidates (Designs 1–5) based on their predicted structural stability and two optimized consensus designs: D_tot, representing the common stabilizing mutations identified across all 1,000 designs, and D_T5, representing the shared stabilizing mutations among the top five designs (**Supplementary Table 1**). The biophysical properties of the designs were assessed to identify the most suitable candidates for experimental validation.

### Computational and experimental characterization of consensus_design spike

Following the initial *in silico* selection, each candidate was subjected to detailed computational analysis to evaluate three key biophysical properties: solubility, structural compactness, and RNA stability. Solubility predictions were performed using Aggrescan3D (A3D), and structural stability was assessed via cavity size analysis using CASTp 3.0, based on the modeled three-dimensional (3D) structure in both software packages. For each candidate protein, codon-optimized RNA sequences with IDT Codon Optimization Tool, were analyzed using RNAfold to assess RNA secondary structure stability and to ensure minimal free energy and stable RNA structures for efficient mRNA translation in host cells. Among the tested designs, D2 demonstrated a balanced combination of these properties, with competitive values for RNA stability, solubility, and structural compactness (**Supplementary Table 1**). These characteristics indicated that D2 (consensus_design Spike; Css_dsg S) was the most suitable candidate for further experimental validation (**Figure 1A**). **Table 1** provides a comparative analysis of these spike variants, confirming that Css_dsg S exhibited superior biophysical characteristics compared to Css S and Control spike (Control S) encoding the pre-fusion-stabilized spike protein from the ancestral SARS-CoV-2 strain. To visualize the spatial distribution of the stabilizing mutations and assess their structural relevance, we mapped the selected mutations onto the 3D structure of the Css_dsg S protein (**Figure 1B**) (26). The NTD and RBD are shown in green and pink, respectively, with the key mutations highlighted. The structural alignment of Css_dsg S and Control S confirmed overall structural convergence, particularly in the NTD and RBD regions, supporting the robustness of the rational design strategy (**Figure 1C**).

**Table 1:** In silico structural stability evaluation of Css_dsg S compared with Css S and Control S.

Spike mRNA	Energy	Solubility	RNA 2D	Cavity
Control S	-702.8	-391.4	-560.8	3521.1
Css S	-1038.7	-340.4	-655.1	1198.7
Css_dsg S	-2084.5	-338.8	-667.8	551.5

Energy: free energy of protein structure from Rosetta relax calculation; Solubility: predicted protein solubility; RNA 2D: minimum free energy of RNA secondary structure; and Cavity: internal cavity volume within the protein. Lower energy and cavity, along with favorable solubility and RNA 2D, indicate improved stability and optimized design of Css_dsg S versus Control S.

To evaluate whether the NTD mutations introduced through *in silico* optimization of Css_dsg S affected its antigenicity, we assessed its binding affinity to the neutralizing monoclonal antibody 4A8, which targets the NTD. Docking simulations and binding affinity estimation revealed that Css_dsg S (ΔG: -15.8 kcal/mol) exhibited a higher binding affinity to 4A8 compared to the original Css S (ΔG: -13.3 kcal/mol), suggesting enhanced immune recognition (**Supplementary Figures 1A, B**). These findings highlight the potential of Css_dsg S for effective immune activation.

Following computational and binding affinity evaluations, the final optimized spike protein sequence (Css_dsg S) was synthesized as mRNA. *In vitro* transcription included key modifications, such as N1-methyl-pseudouridine substitution and co-transcriptional capping, to enhance mRNA stability and translation efficiency. The synthesized mRNA was transiently transfected into HEK293T cells using a liposome-based delivery system. Western blot analysis confirmed successful translation and improved expression of the designed spike protein 24 h after transfection (**Supplementary Figures 1C, D**). Collectively, these results support the superior structural properties of Css_dsg S, establishing it as an optimal candidate for vaccine development.

### Immunogenicity of *in silico*-designed spike mRNA vaccine in BALB/c mice

**Figure 2:**
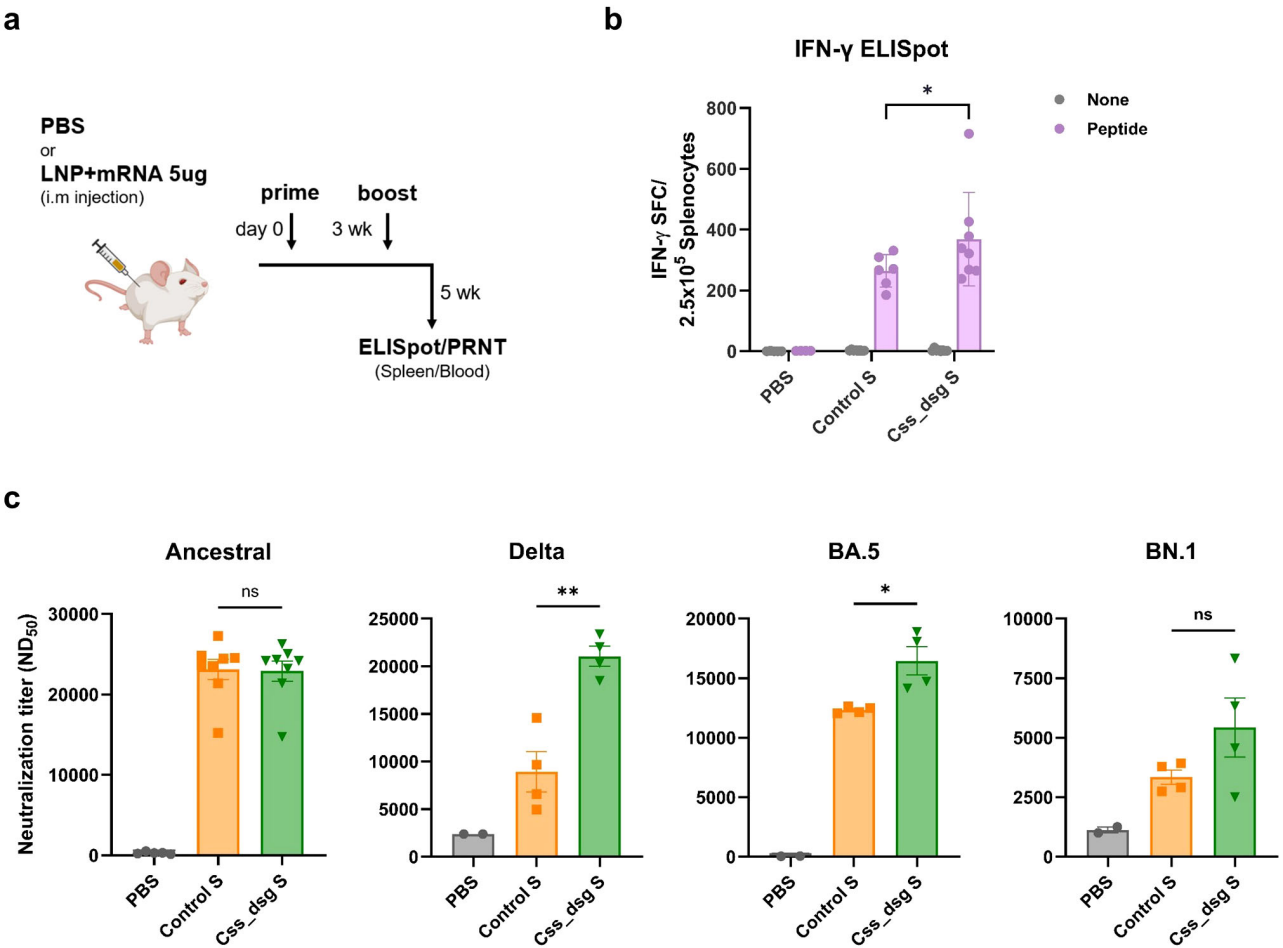
*In silico* design of spike mRNA vaccine induces potent cellular and humoral immune responses against variant of concerns in BALB/c mice. **(a)** Immunization and bleed schedule for BALB/c mice. Groups of mice (n=8) were vaccinated intramuscularly with two doses of Control spike, *in silico* spike, or PBS buffer at 3-week intervals. **(b)** Total splenocytes of immunized mice were collected 2 weeks after the booster vaccination. Cells were re-stimulated with the ancestral spike peptide pool, and splenocytes secreting IFN-γ were measured using ELISpot assay. *P*-values were determined using two-way ANOVA and Turkey’s multiple comparison, ns, not significant, *p < 0.05, **p < 0.01. **(c)** Sera of the vaccinated mice were collected 2 weeks after the second vaccination and the neutralizing antibody titers in the sera were analyzed via a plaque reduction neutralization test (PRNT) using the ancestral, Delta, BA.5, and BN.1 SARS-CoV-2 viruses. P-values were determined using one-way ANOVA with Dunnett’s test, ns, not significant, *p < 0.05, **p < 0.01.

To evaluate the immunogenicity of the *in silico*-designed Css_dsg S antigen, we generated a nucleoside-modified mRNA vaccine formulated with lipid nanoparticles (LNPs). Phosphate-buffered saline (PBS) and a Control S vaccine served as controls. BALB/c mice received two doses of either the Css_dsg S vaccine, Control S vaccine, or PBS, with a 3-week interval between doses (**Figure 2A**).

Two weeks after the booster dose, total splenocytes were harvested and re-stimulated with an ancestral spike peptide pool to assess antigen-specific cellular immune responses. Mice immunized with Css_dsg S exhibited a significantly higher frequency of IFN-γ-secreting splenocytes than those in the Control S group (**Figure 2B**), indicating a robust cellular immune response. Neutralizing responses against the ancestral virus were comparable between Css_dsg S and Control S. However, Css_dsg S vaccination induced significantly higher neutralizing antibody titers against SARS-CoV-2 variants, including Delta and Omicron BA.5 variants, not against the Omicron BN.1 variant, compared to Control S (**Figure 2C**). A separate analysis that included the Css S group (**Supplementary Figure 2**) further supported this trend, showing increased neutralizing antibody levels in Css S and Css_dsg S compared to Control S, with Css_dsg S eliciting the highest response against Delta and Omicron BA.5. These findings highlight the enhanced immunogenicity of Css_dsg S against the SARS-CoV-2 variants.

### Protective efficacy of Css_dsg S in K18-hACE-2 transgenic mice

**Figure 3:**
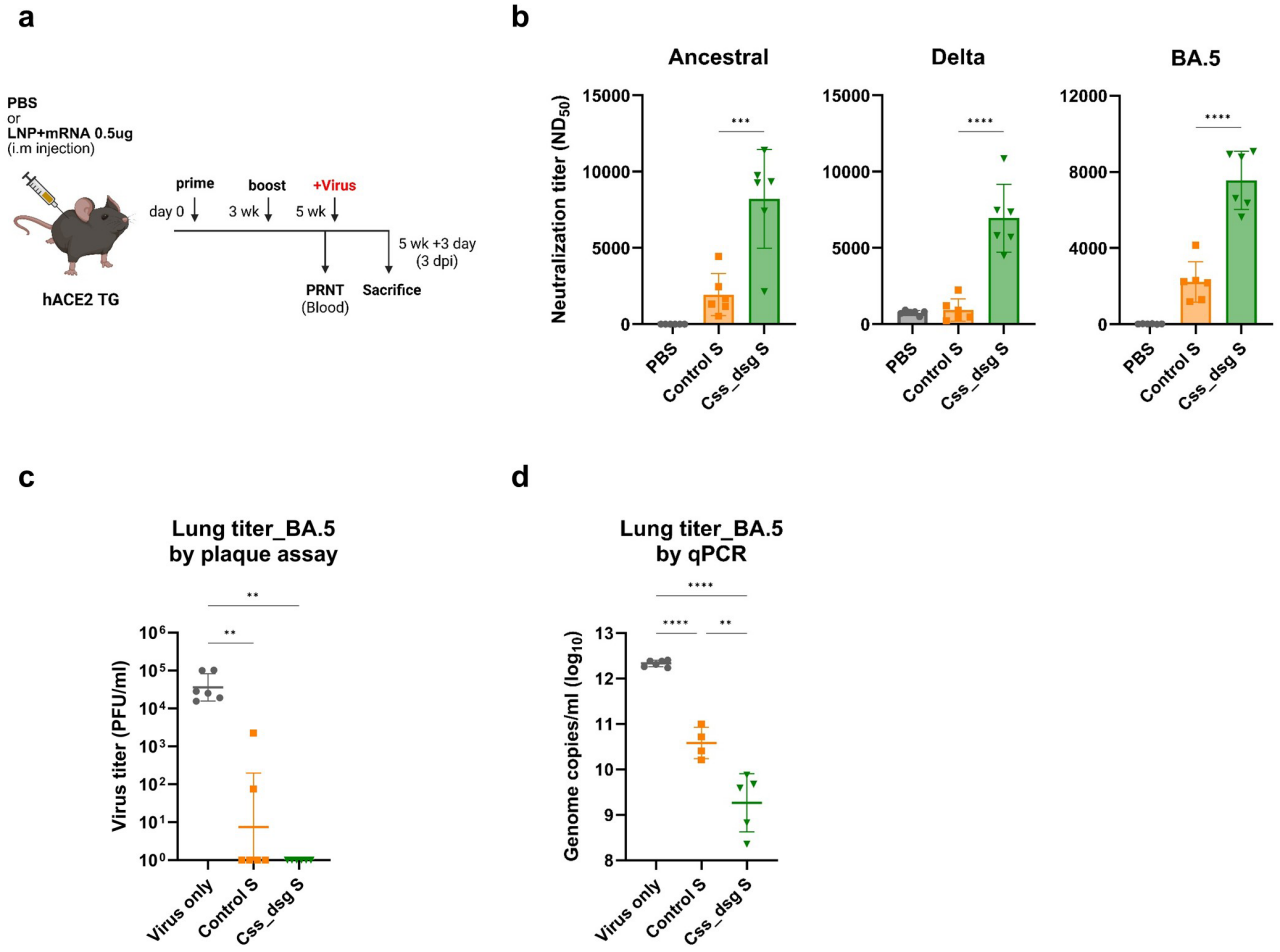
*In silico* design of spike mRNA vaccine elicits a superior protective efficacy against SARS-CoV-2 variant challenge in K18-hACE-2 transgenic mice. **(a)** Immunization, bleed, and challenge schedule for K18-hACE-2 mice. K18-hACE-2 mice were vaccinated intramuscularly with two doses of Control spike, *in silico* spike, or PBS buffer at 3-week intervals. The mice were challenged after 2 weeks using the SARS-CoV-2 virus against the ancestral virus (1.25 × 10^4^ PFU), Delta (8 × 10^4^ PFU), and Omicron BA.5 (9 × 10^4^ PFU) variants. **(b)** Neutralizing antibody titers in the serum of the vaccinated mice were analyzed via a PRNT using the SARS-CoV-2 virus against the ancestral, Delta, and Omicron BA.5 virus. P-values were determined using one-way ANOVA with Dunnett’s test, ns, not significant, ***p < 0.001, ****p < 0.0001. **(c, d)** Infectious viral titers detected using plaque **(c)** and qRT-PCR assays **(d)** in the lung at 3 dpi with 9 × 10^4^ PFU Omicron BA.5 virus.

The protective efficacy of the Css_dsg S mRNA vaccine was evaluated in K18-hACE2 transgenic mice, a model that is highly susceptible to SARS-CoV-2 infection. Mice were vaccinated intramuscularly with the Css_dsg S vaccine, Control S vaccine, or PBS, followed by a 3-week interval before viral challenge (**Figure 3A**).

In BALB/c mice, neutralizing antibody responses against the original virus showed no significant difference between Css_dsg S and Control S, possibly due to saturation at the 5 μg dose, where neutralizing antibody levels may have plateaued, thereby masking group differences. To better assess neutralizing responses, the vaccine dose was reduced to 0.5 μg in K18-hACE2 mice. Two weeks after vaccination, the mice were challenged with the original virus S (1.25 × 10^4^ Plaque Forming Units [PFU]) or SARS-CoV-2 variants, including Delta (8 × 10^4^ PFU) and Omicron BA.5 (9 × 10^4^ PFU). One day before viral challenge, serum samples were collected and analyzed using a plaque reduction neutralization (PRNT) assay to determine neutralizing antibody levels against the original virus, Delta, and Omicron BA.5. Mice vaccinated with Css_dsg S exhibited significantly higher neutralizing antibody levels against the original virus and all tested variants than mice that received Control S (**Figure 3B**).

To assess clinical outcomes, changes in body weight and survival rates were monitored for 14 days after infection. In the PBS control group, 100% mortality was observed on days 9 (original virus) and 7 (Delta) after infection (**Supplementary Figure 3A**). In contrast, both vaccinated groups (Control S and Css_dsg S) showed 100% survival until the end of the experiment. Mice in the vaccinated groups showed no significant weight loss during the first 2–3 days after infection (**Supplementary Figure 3B**). Notably, after the Delta challenge, mice vaccinated with Css_dsg S showed faster weight gain, starting from day 2 after infection, whereas the Control S group began gaining weight from day 4. This faster recovery of body weight suggests that Css_dsg S provides more effective immune protection against Delta infections.

To evaluate viral replication, lung tissues from mice challenged with Omicron BA.5 were collected 3 days after infection (dpi). Infectious viral titers were determined using plaque assays and qRT-PCR. Mice vaccinated with Css_dsg S had significantly lower viral loads in the lungs than mice receiving Control S (**Figures 3C, D**). Collectively, these results demonstrate that the *in silico*-designed Css_dsg S mRNA vaccine provides a robust immune response and effective viral control against SARS-CoV-2 variants.

### Design and validation Omicron-based *in silico* spike antigens

**Figure 4:**
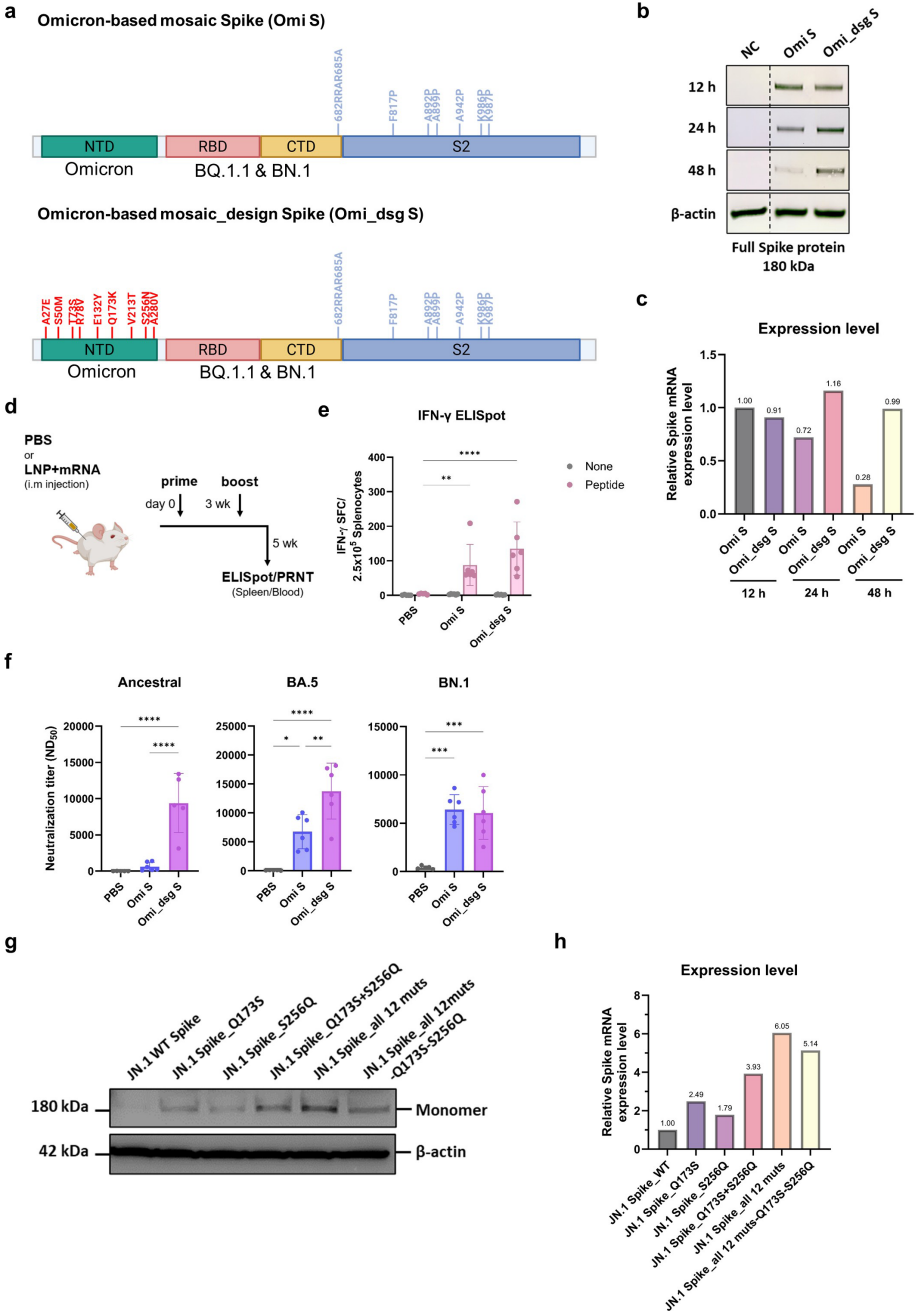
Design and optimization of a omicron-based mosaic spike antigens with NTD stabilizing mutations. **(a)** Schematic of the Omi S and Omi_dsg S spike antigen constructs. Omi S is a mosaic antigen composed of NTD and RBD sequences from Omicron variants and an S2 region from Css_dsg S. Omi_dsg S was further optimized for structural stability and codon usage. **(b)** Western blot analysis of spike protein expression in HEK293T cells transfected with *in vitro*-transcribed Omi S or Omi_dsg S mRNA. Omi_dsg S showed enhanced and prolonged expression at 48 h after transfection. **(c)** Relative spike protein expression levels with quantitative values of western blot analysis. The intensity of the bands in **(b)** was quantified to the expression of Omi S at 12 h using ImageJ software. **(d)** Schematic of the mouse immunization schedule. BALB/c mice were immunized with PBS, Omi S, or Omi_dsg S mRNA vaccines. **(e)** IFN-γ ELISpot analysis of splenocytes from vaccinated mice. **(f)** Neutralizing antibody titers measured against SARS-CoV-2 variants at 2 weeks after immunization. Omi_dsg S induced significantly higher neutralization titers than Omi S. P-values were determined using one-way ANOVA with Dunnett’s test, ns, not significant, *p < 0.05, **p < 0.01, ***p < 0.001, ****p < 0.0001. **(g)** Western blot analysis of spike protein expression from JN.1-based constructs transfected into HEK293T cells. Q173S and S256Q mutations, alone or in combination, showed increased expression levels compared to wild-type JN.1 spike. Omission of these mutations from a 12-mutation NTD design reduced expression, supporting their functional role as key stabilizing residues. **(h)**, Relative spike protein expression levels with quantitative values of western blot analysis. The intensity of the bands in **(f)** was quantified to the expression of JN.1 WT Spike using ImageJ software.

To address the challenges posed by Omicron variants, we extended the computational design strategy validated in Css_dsg S to develop Omicron-based spike antigens. A chimeric sequence was generated as an Omicron-based mosaic spike, incorporating the NTD and RBD sequences derived using a POMA approach. The NTD consensus was based on conserved features of Omicron variants, and the RBD consensus integrated sequences from BQ.1.1 and BN.1. The S2 domain was retained in Css_dsg S to maintain its structural stability (Omi S in **Figure 4A**).

Advanced structural modeling using AlphaFold2 and pSUFER identified suboptimal regions within the NTD, which were refined to enhance protein stability. The rational design pipeline, including FuncLib analysis and biophysical property evaluation, was consistently applied and the final construct was selected from seven candidate designs (**Supplementary Tables 2**, **3**). To further improve RNA stability and translation efficiency, we used LinearDesign to generate an optimized variant with superior RNA-folding characteristics and codon usage (Omicron-based mosaic_desgin spike; Omi_dsg S in **Figure 4A**). *In vitro*-transcribed Omi S and Omi_dsg S mRNAs were transfected into HEK293T cells, where the optimized design showed significantly higher and sustained protein expression 48 h after transfection, compared to the unoptimized Omi S (**Figures 4B, C**). Moreover, HADDOCK docking analysis and binding affinity estimation revealed that Omi_dsg S (ΔG: -14.8 kcal/mol) had a stronger predicted interaction with the NTD-targeting antibody 4A8 than Omi S (ΔG: -13.6 kcal/mol), supporting preserved and potentially enhanced antigenicity (**Supplementary Figure 4**).

To evaluate immunogenicity, BALB/c mice were intramuscularly immunized with Omi S or Omi_dsg S mRNA vaccines in LNPs, followed by a boost at week 3 (**Figure 4D**). At week 5, IFN-γ ELISpot analysis showed that Omi_dsg S tended to induce higher peptide-specific T cell responses than Omi S (**Figure 4E**). Neutralization assays revealed that Omi_dsg S elicited substantially higher antibody titers than Omi S against both the ancestral virus and BA.5 variant, while titers against BN.1 were comparable between the two vaccine groups (**Figure 4F**). These results suggest that structural optimization of Omi_dsg S may modestly enhance cellular immunity and more strongly augment humoral immunity against specific variants.

Notably, two critical stabilizing residues in NTD - Q173 and S256 were consistently identified across both optimized designs. Css_dsg S contained Q173S and S256Q, whereas Omi_dsg S had Q173K and S256N. These residue numbers were based on the ancestral spike protein and correspond to Q170 and S253 in the Css_dsg S and Omi_dsg S sequences, respectively (**Supplementary Figure 5**, **Supplementary Table 4**). We hypothesized that these mutations were selected based on their predicted capacity to enhance NTD stability and spike expression. To validate this, we introduced all 12 NTD mutations from Css_dsg S and Omi_dsg S into the spike protein of the recently emerged JN.1 variant, in which the corresponding residues are Q171 and S253 (**Supplementary Figure 5**). The following experimental groups were generated: JN.1 wild-type spike (JN.1 S), JN.1 S_173S, JN.1 S_S256Q, JN.1 S_Q173S + S256Q, JN.1 S_all 12 NTD mutations and JN.1 S_all 12 NTD mutations – (Q173S + S256Q). These constructs were transiently transfected into HEK293T cells, and protein expression was assessed 24 h after transfection using western blot analysis (**Figure 4G**). Notably, the JN.1 spike harboring the combined Q173S + S256Q mutations exhibited significantly enhanced protein expression compared to the WT spike. Similarly, the spike protein containing all 12 NTD mutations demonstrated enhanced expression; however, when the two critical mutations (Q173S + S256Q) were excluded from the full 12-mutation NTD (all 12 NTD Mutations – [Q173S + S256Q]), protein expression significantly decreased, indicating that enhanced stability and expression were primarily driven by these two key sites (**Figure 4H**). These results confirmed that Q173 and S256 were identified as recurring stabilizing sites across the Css_dsg S and Omi_dsg S designs, indicating their potential as universal NTD-stabilizing hotspots. Other mutations introduced in a structure-guided manner may exert their effects in a context-dependent fashion, synergizing with the antigen-specific structural landscape to improve protein integrity and immunogenicity. This finding aligns with our *in silico* predictions and further validates the importance of these stabilization sites for optimized spike antigen design.

### Evaluation of the protective efficacy of the bivalent Css_dsg S and Omi_dsg S mRNA vaccine

**Figure 5:**
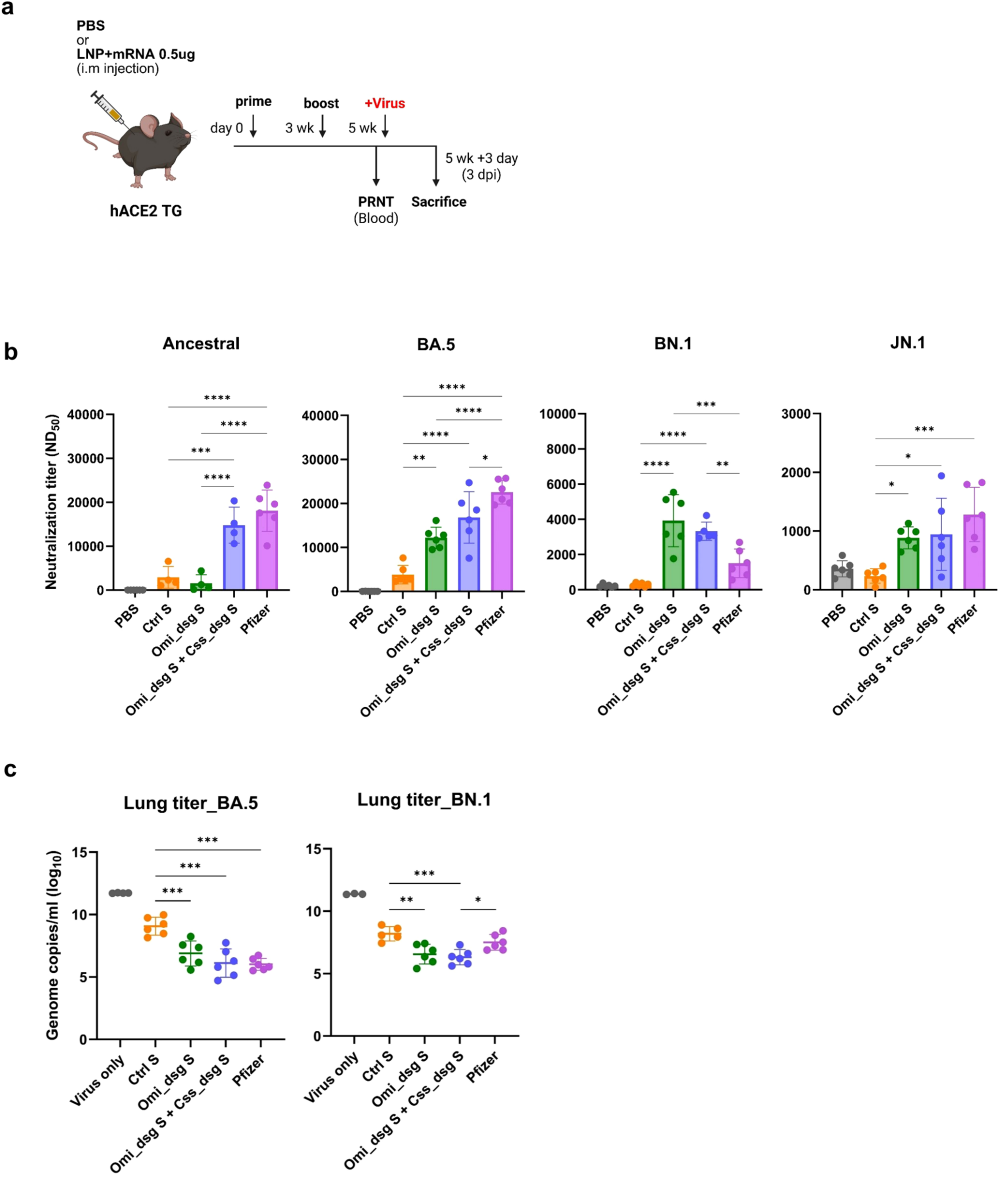
Protective efficacy of the bivalent Css_dsg S + Omi_dsg S mRNA vaccine. **(a)** Immunization and challenge schedule for K18-hACE2 transgenic mice. Mice received either monovalent (Control S or Omi_dsg S) or bivalent (Css_dsg S + Omi_dsg S) vaccines before SARS-CoV-2 challenge. The BNT162b2 bivalent mRNA vaccine (Pfizer-BioNTech, BA.4-5) was included as a control. **(b)** Neutralizing antibody titers measured against the ancestral strain, Omicron BA.5, BN.1 and JN.1 variants in mice administered monovalent or bivalent vaccines. **(c)** Lung viral load at 3 dpi, measured via a plaque assay against BA.5 (c) and BN.1 (d), showing significantly reduced viral titers in the bivalent vaccine group compared to Control S. P-values were determined using one-way ANOVA with Dunnett’s test, ns, not significant, *p < 0.05, **p < 0.01, ***p < 0.001, ****p < 0.0001.

To achieve comprehensive protection against SARS-CoV-2, we evaluated a bivalent mRNA vaccine formulation comprising Css_dsg S and Omi_dsg S. hACE2 transgenic mice were vaccinated with either a monovalent (Control S or Omi_dsg S) or bivalent (Css_dsg S + Omi_dsg S) formulation and subsequently challenged with the original virus, Omicron BA.5, and BN.1 variants (**Figure 5A**). The bivalent BNT162b2 mRNA original/Omicron BA.4–5 vaccine (Pfizer-BioNTech) was used as an FDA-approved control. Analysis of neutralizing antibody responses revealed that the bivalent Css_dsg S + Omi_dsg S vaccine elicited significantly higher neutralizing titers against the original strain (S), Omicron BA.5, BN.1, and JN.1 compared to the monovalent vaccines, including Control S and Omi_dsg S (**Figure 5B**). Notably, neutralizing titers in the bivalent group were comparable to those observed in the Pfizer control group for most variants, with statistically significant enhancement against BN.1. For survival monitoring, all mice in the PBS group succumbed to infection on day 8, whereas all vaccinated groups, including those receiving monovalent and bivalent vaccines, showed 100% survival (**Supplementary Figure 6A**). Body weight monitoring indicated that mice in all vaccinated groups, except those receiving Control S, experienced minimal to no weight loss during the first 4 days after infection, whereas substantial weight loss was observed in the PBS groups (**Supplementary Figure 6B**). Lung viral load analysis conducted 3 days after the challenge showed that the bivalent Css_dsg S + Omi_dsg S vaccine effectively reduced viral loads in the lungs of vaccinated mice. In the Omicron BA.5 challenge, the viral loads in the bivalent vaccine group were comparable to those in the Pfizer control group, indicating equivalent viral suppression. Moreover, during the BN.1 challenge, the bivalent vaccine achieved significantly lower lung viral loads than the Pfizer control group, demonstrating superior viral suppression (**Figure 5C**). These results demonstrate that the bivalent Css_dsg S + Omi_dsg S vaccine provides broad and enhanced protection against SARS-CoV-2, including the Omicron variants, supporting its potential as a versatile and broadly protective mRNA vaccine strategy.

## Discussion

This study demonstrates the feasibility of computationally designed spike protein antigens for SARS-CoV-2 mRNA vaccines, yielding significant advancements in structural stability, immunogenicity, and cross-variant protection. By leveraging advanced *in silico* techniques, we successfully identified suboptimal stability sites within the spike protein and introduced energetically favorable mutations to enhance antigenicity. These findings highlight the potential of computational frameworks for the development of next-generation vaccines against rapidly evolving pathogens.

The design began with a POMA approach, which generated a consensus sequence by integrating conserved features from multiple SARS-CoV-2 variants, comprising from the ancestral to Delta strain for Css_dsg S or Omicron variants for Omi_dsg S. This strategy ensured broad antigenic coverage across diverse viral strains, providing a solid foundation for further optimization. Advanced structural modeling using AlphaFold2 enabled the precise visualization of the S1 subunit of the spike protein, revealing important structural regions susceptible to instability. Using pSUFER and FuncLib, we systematically identified energetically suboptimal regions within the NTD and introduced stabilizing mutations predicted to enhance structural integrity. These computationally guided modifications were further refined through iterative analysis, ensuring that only the most favorable mutations were retained. This rational design approach enabled the development of novel spike protein antigens with enhanced stability and expression, providing a versatile framework for rapid antigen optimization.

The immunogenicity of a vaccine is determined by multiple factors, including the structural stability of the antigenic proteins and accessibility of epitopes for antibody binding. To assess these properties, we used AlphaFold2-based structural modeling and af2rank analysis, which revealed that the computationally designed antigen Css_dsg S exhibited enhanced structural stability compared to its parental form, Css S. This improvement in stability was further supported by antigen-antibody binding analysis using the HADDOCK server, where the Css_dsg S-NTD and 4A8 complex exhibited superior stability compared to the Css S-NTD and 4A8 complexes. To investigate their potential for antibody recognition, we analyzed the surface accessibility of the epitopes using PyMOL. The results confirmed that antigen Css_dsg S (10810.5 Å^2^) exhibited greater epitope exposure than Css S (10504.1 Å^2^), and antigen Omi_dsg S (10913.5 Å^2^) displayed higher surface accessibility than Omi S (10768.7 Å^2^). Increased epitope exposure is critical for effective antibody recognition and subsequent antibody generation, suggesting that these antigens are likely to induce stronger immune responses. Collectively, these findings validate our computational design strategy for the optimization spike protein antigens. The enhanced structural stability and epitope accessibility achieved through rational design are expected to directly contribute to the superior immunogenicity of the Css_dsg S and Omi_dsg S antigens observed in this study.

*In vivo* evaluations in BALB/c and K18-hACE2 transgenic mice confirmed the immunogenic efficacy of the computationally designed spike mRNA vaccines. Mice vaccinated with Css_dsg S developed higher T cell responses and elevated titers of neutralizing antibodies against SARS-CoV-2 variants, including Delta and Omicron BA.5 variants. In addition, a notable reduction in viral loads in the lung tissues was observed within 3 days post-challenge, underscoring the protective effect of the vaccine. Similarly, vaccination with Omi_dsg S led to enhanced neutralizing antibody responses and T cell activation compared with its unoptimized counterpart, Omi S, further supporting the effectiveness of the structure-guided design. These findings align with emerging evidence that computationally optimized antigens may elicit more potent and variant-resilient immune responses than traditionally designed antigens.

Beyond the *in vivo* validation of immunogenicity, we investigated the molecular basis of this enhanced immune response through *in silico* analysis. Our *in silico* design strategy consistently identified two key stabilization sites, Q173 and S256, within the NTD of the spike protein, which were introduced into both Css_dsg S and Omi_dsg S. These sites emerged as critical stabilization points, and context-dependent mutations (Q173S and S256Q in Css_dsg S, and Q173K and S256N in Omi_dsg S) significantly enhanced protein stability and expression. Experimental analysis using the JN.1 spike supported this observation, in which introducing Q173S and S256Q markedly improved protein expression, whereas removing these two mutations from the 12 introduced mutations led to a significant decrease. These findings suggest that Q173 and S256 serve as universal stabilization sites within the NTD, providing rational and strategic targets for optimizing spike protein antigens across variants. In addition to these two critical NTD sites, other mutations were introduced as part of the overall structure-guided antigen design. While these additional mutations may also contribute to antigen stability or immunogenicity, their individual roles were not systematically dissected in this study. As the NTD is highly variable and escape-prone, stabilizing mutation at Q173 and S256 likely reduce conformational lability, thereby enhancing cross-variant neutralization and protection. Notably, these NTD mutations, located in conserved supersites associated with neutralizing antibody induction and immune activation, further support the versatility of this approach (27, 28). The Css_dsg S antigen, derived from conserved sequences of the ancestral and Delta strains, demonstrated broad protection against Omicron, whereas the Omi_dsg S antigen, specifically tailored for Omicron variants, further validated the effectiveness of this design strategy. These findings underscore the potential of rationally optimized NTD to enhance antigen stability and cross-variant protection.

Further investigation is needed to determine whether Q173 and S256, as well as other introduced mutations, play similar stabilizing roles in spike proteins derived from additional SARS-CoV-2 variants. Although the enhanced antigenicity and broad immune responses observed in this study may be partly attributed to the increased protein stability conferred by these mutations, the introduced structural modifications as a whole may also have altered conformational epitopes, thereby improving immune recognition. Determining whether these effects are primarily driven by thermodynamic stabilization or by epitope remodeling resulting from the introduced structural modifications will be critical for guiding future antigen design strategies. Notably, the stabilizing effects of Q173 and S256 appeared to be consistent regardless of the presence of furin cleavage site mutations or pre-fusion-stabilizing 6P modifications, suggesting that these residues contribute to spike stability through an independent and broadly applicable mechanism. Although our *in silico* design and experimental data underscore the significance of Q173 and S256 as key stabilization sites within the NTD, the structural basis of this effect remains speculative. Cryo-electron microscopy analysis can provide direct structural insights into how these mutations affect NTD architecture, epitope exposure, and overall trimer integrity. Such investigations will help bridge the gap between computational predictions and empirical structural validation.

The bivalent vaccine formulation (Css_dsg S + Omi_dsg S) elicited broad immune responses against Omicron variants while maintaining neutralization against earlier SARS-CoV-2 strains. Although each antigen was administered at half the dose compared to the corresponding monovalent vaccines, the bivalent formulation induced immune responses that were comparable to those achieved by monovalent vaccination, supporting a synergistic contribution of Css_dsg S and Omi_dsg S in shaping cross-variant immunity.

Importantly, the magnitude of neutralizing activity differed depending on the target variant. While the bivalent formulation demonstrated statistically significant enhancement of neutralizing activity and viral suppression against BN.1, neutralizing titers against BA.5 were significantly lower than those observed with the BA.4/5-adapted comparator vaccine. This observation underscores that the benefit of the bivalent design is not uniform across all variants, but instead reflects the antigen composition and variant coverage targeted during vaccine design. Notably, the comparator bivalent vaccine was optimized against the original strain and BA.4/5 lineages and was not designed to target BN.1, whereas the bivalent vaccine described in this study incorporated antigenic features intended to improve cross-variant coverage, including emerging Omicron sublineages.

Together, these findings highlight the potential advantages of multi-variant antigen formulations in broadening immune coverage without increasing the total mRNA dose, while also emphasizing the importance of aligning antigen design with the intended variant landscape. Future studies will be required to further assess the protective efficacy of Omi_dsg S against additional emerging variants and to refine antigen compositions that balance variant-specific potency with cross-variant breadth. In addition, incorporating the identified NTD-stabilizing mutations, such as Q173S and S256Q, into future variant-specific designs may provide further insight into how these mutations influence antibody–antigen interactions and overall immunogenicity.

This study presented a computationally driven framework for designing mRNA vaccine antigens with broad-spectrum efficacy and enhanced immunogenicity. By focusing on the structural stability and RNA optimization, we demonstrated the feasibility of developing adaptable and effective vaccine candidates. The robust immune responses and protection observed against Omicron variants underscore the potential of this approach for combating SARS-CoV-2 and other rapidly evolving pathogens. Our findings underscore the utility of rational design for the development of next-generation mRNA vaccines and lay the groundwork for future antigen strategies targeting emerging SARS-CoV-2 variants and other rapidly mutating pathogens.

## Methods

### Consensus and mosaic sequences generation

The initial template antigen sequence used in this study was obtained from Korea University. This sequence was used as the starting point for the structural modeling and subsequent simulations. A consensus sequence was generated using the POMA approach, incorporating conserved sequence features across representative SARS-CoV-2 variants selected to capture genetic diversity spanning from the ancestral strain to Delta-related lineages. To optimize the pre-fusion conformation, structure-guided stabilizing mutations, including 6P substitutions in the S2 domain and cleavage site modifications, were introduced. The resulting antigen sequence was designated Css S. The template sequence used for the omicron-based mosaic spike (Omi S) was also developed by Korea University. This sequence was composed of three regions (NTD, RBD, and the S2 subunit), each constructed from the spike protein of distinct variants chosen based on their phylogenetic relationships, as identified using the POMA strategy. The NTD consensus was generated from conserved sequence features of Omicron variants, and the RBD consensus was derived from preserved sequences of BQ.1.1 and BN.1 variants. The S2 subunit contained the C-terminal Domain (CTD) of S1, which originated from the sequence of the ancestral strain, and 6P-stabilization and cleavage site substitutions were applied.

### Structure modeling and computational design

The S1 subunit of spike protein was subjected to structure prediction, and structure modeling was performed on the open-source platform AlphaFold2-Colab on Google (https://colab.research.google.com/github/sokrypton/ColabFold/blob/main/AlphaFold2.ipynb) with the amber relaxed (29). The prediction was made using default parameters. With the predicted model, a combined pSUFER–FuncLib approach was employed to design multi-mutant variants (30, 31). pSUFER provides an available site set on energetically suboptimal regions through computational saturation mutagenesis, and then FuncLib is designed for multipoint mutations at given sites in pSUFER through evolutionary analysis and Rosetta energy calculations based on the modeled 3D structure of S1. Finally, a total of 1,000 designs was generated and ranked by predicted stability, and the resulting models were visually inspected in PyMOL (32).

### Computational analysis of biophysical properties for designed candidates

To select rational candidates among the 1,000 designs, we first chose a candidate set of the top five designs (D1–D5) and two consensus designs for all designs and for the top five designs. For the final *in silico* candidate selection, we considered three properties; solubility, cavity (package), and stability of RNA 2D structure. For mRNA 2D structure analysis, nucleotide sequences corresponding to each candidate were first generated using the Integrated DNA Technologies (IDT) codon optimization tool to maximize expression efficiency in host for initial in silico design. Solubility, cavity, and RNA 2d structure are important factors for protein expression and structural stability. A3D (33) is a computational tool designed to predict the solubility of proteins in aqueous solutions based on their amino acid sequences. The program analyzes the physicochemical properties of the protein to estimate its solubility, considering the relationship between solubility and structural stability, and assesses the likelihood of stable dissolution of the protein in solution. CASTp-3.0 (34) is a software tool that analyzes the 3D structure of proteins to identify cavities within the complex. It evaluates the size, shape, and location of cavities, both on the surface and interior of the protein, providing critical insights into the functional regions and binding properties of the protein. These analyses provided valuable data for assessing the structural stability of proteins. RNAfold (35) is a computational tool that predicts the secondary structures and corresponding energy states of a given RNA sequence. Based on the principle of minimum free energy, it determines the optimized secondary structure of RNA and evaluates its structural stability. RNA stability plays a critical role in understanding protein-RNA interactions, which can affect the functional stability of the protein. To further improve RNA 2D structural stability, LinearDesign (36) was later applied for omicron-based chimeric design. LinearDesign simultaneously performs RNA secondary structure optimization and synonymous codon assignment, generating optimized mRNA sequences for computational evaluation.

### *In silico* antigen–antibody complex modeling and scoring

Putative complexes between each spike‐NTD variant (parental, Css_dsg S or Omi_dsg S) and the NTD-directed monoclonal antibody 4A8 were generated with the AlphaFold2-multimer implementation (37) in AlphaFold2 (default settings) (9). Afterward, the top-ranked model for each pair was refined in HADDOCK (38), and binding free energy (ΔG_bind) was calculated with the PRODIGY (39) web server. Complex quality was assessed using af2rank (40) and MolProbity (41). All the tools were run using the recommended default parameters.

### Preparation of mRNA-LNP vaccines

A human codon-optimized coding sequence (IDT codon optimization tool) was synthesized and cloned into an mRNA production plasmid. A linearized DNA template containing the open reading frame flanked by 5′ and 3′ untranslated regions (UTRs) (5′ UTR: GGGAAAUAAGAGAGAAAAGAAGAGUAAGAAGAAAUAUAAGAGCCACC; and 3′ UTR: UGAUAAUAGGCUGGAGCCUCGGUGGCCAUGCUUCUUGCCCCUUGGGCCUCCCCCCAGCCCCUCCUCCCCUUCCUGCACCC GUACCCCCGUGGUCUUUGAAUAAAGUCUGA) and 120-nucleotide poly(A) tails was produced using PCR amplification. mRNAs were synthesized *in vitro* using T7 RNA polymerase-mediated transcription with complete replacement of uridine by N1-methyl-pseudouridine-5′-triphosphate (m1Ψ, N-1081, TriLink). Capping of the *in vitro*-transcribed mRNAs was performed co-transcriptionally using the trinucleotide cap1 analog (m7(3’OMeG)(5’)ppp(5’)(2’OMeA)pG, N-7413, TriLink). After *in vitro* transcription, the DNA template was removed using DNase, and the final mRNA product was purified using RNeasy kit (74104, QIAGEN). RNA integrity was analyzed using automated electrophoresis (Agilent TapeStation, Agilent Technologies). RNA concentration was spectrophotometrically quantified, and the sample was stored at -80°C prior to LNP formulation. LNPs were prepared using a NanoAssembly Benchtop Instrument (Precision Nanosystems Inc.). Ionizable lipids (SM-102, 2089251-47-6, SINOPEG), cholesterol (C3045-100G, Sigma-Aldrich), distearoylphosphatidylcholine (DSPC, 850365P, Avanti), and PEG-lipid (C16 PEG2000 Ceramide, 880180P-200 mg, Avanti) were dissolved in ethanol, and the mRNA was diluted in 10 mM citrate (pH 3). The molar ratio of the lipid components was as follows: SM-102:DSPC:cholesterol:PEG2000 = 50:10:38.5:1.5. The final ionizable lipid:RNA weight ratio was 10:1 and the final volume ratio was 1:3. LNPs were formulated by microfluidic mixing of the prepared solutions at a flow rate of 12 mL/min. The resulting LNPs were diluted in a 40-fold volume of 1× PBS and concentrated via ultrafiltration (Amicon^®^ Ultra-15 Centrifugal Filter Unit, UFC9010). The physical properties of mRNA-loaded LNPs were characterized. The hydrodynamic size was measured via Dynamic Light Scattering using a Malvern Nano-ZS Zetasizer (Malvern Instruments Ltd.) to confirm the mean particle diameter and polydispersity index. RNA concentration and encapsulation efficiency were confirmed using RiboGreen Assay (Thermo Fisher Scientific, Waltham, MA, USA). The recombinant SARS-CoV-2 spike protein was composed of a consensus sequence from 537 isolates belonging to the Delta variant in Korea, containing six proline mutations for stability. The protein was produced in a mammalian expression system using the CHO-S cell line and purified using a HisTrap™ FF Crude column.

### Mice and immunization

Female BALB/c mice aged 4–6 weeks were purchased from Ja Bio and Samtako Bio, Korea. The mice were housed and bred in a specific pathogen-free mouse facility with controlled temperature, humidity, and light cycle. The mice were housed in the Animal Biosafety Level 2 facility at the National Institute of Health, South Korea. The mice were immunized intramuscularly with 50 ul of either PBS (mock) or mRNA-LNP (5 µg) at weeks 0 (prime) and 3 (boost). Two weeks after the booster vaccination, all mice were anesthetized using intraperitoneal administration of 10 mg/kg Rompun and 100 mg/kg Ketamine. Blood was collected via facial vein puncture under anesthesia. Subsequently, the mice were euthanized by CO_2_ inhalation, and the spleen was collected. For the protective immunity test, K18-hACE2 transgenic mice [B6.Cg-Tg(K18-ACE2)2Prlmn/J)] were used animal models for SARS-CoV-2 challenge. The mice were housed in the Animal Biosafety Level 3 facility. The mice were immunized with two shots of either PBS, 0.5 μg of mRNA-LNP or Comirnaty Original/Omicron BA.4–5 vaccine (Pfizer, provided for research purposes). All prime and booster doses were administered at a 3-week interval. Two weeks after the booster immunization, the transgenic mice were intranasally challenged with 30 µL containing the ancestral virus (1.25 × 10^4^ PFU), Delta (8 × 10^4^ PFU), or Omicron BA.5 (9 × 10^4^ PFU) variant. Mice were euthanized on the third day after infection to measure viral titers in the lung tissues. For sample collection, the mice were anesthetized via intraperitoneal administration of 10 mg/kg Rompun and 100 mg/kg Ketamine. The animal protocols used in this study were reviewed and approved by the Institutional Animal Care and Use Committee of the Korea Centers for Disease Control and Prevention (KDCA-IACUC-23-013, KDCA-IACUC-24-028, and KDCA-IACUC-25-024).

### Cell and virus harvest

Vero E6 cells were grown in Dulbecco’s Modified Eagle Medium supplemented with 10% heat-inactivated fetal bovine serum and 1% penicillin/streptomycin. Cells were cultured at 37 °C under 5% CO_2_ in an incubator. The first human-isolated SARS-CoV-2 strain in Korea (BetaCoV/Korea/KCDC03/2020, NCCP 43326) was passaged and titrated as PFU in Vero E6 cells. All experiments involving live viruses were conducted in a biosafety level 3 facility following the recommended safety precautions and measures.

### Virus titration in infected lung tissue

Lung tissue samples from infected mice were homogenized two times at 8000 rpm for two cycles of 20 s, 10 s break between cycles, following the Precellys animal tissue protocol (Precellys Lysing Kit, CK28R, 2 mL). The supernatant from the homogenized samples was serially diluted tenfold in serum-free medium. Vero E6 cells were seeded in 12-well plates (2 × 10^5^ cells/well) and infected with the diluted lung supernatant. The plates were incubated at 37 °C for 1 h. After incubation, the cells were overlaid with 1 mL of 1.2% agarose overlay medium and further incubated for 2 days at 37 °C. Following incubation, the plaques were visualized by staining with crystal violet solution, and the number of plaques was counted.

### Plaque reduction neutralization test

PRNT was performed to analyze neutralizing antibodies to the mRNA vaccine. PRNT_50_ were determined using the highest serum dilution that inhibited >50% of the number of plaques in the absence of the test serum. Serum samples from 5 weeks after the first vaccination were heat-inactivated for 30 min at 56 °C prior to use. Serum samples prepared from the immunized mice were serially diluted from 1:10 to 1:5120 in a 2% fetal bovine serum medium. Virus–serum mixtures were prepared by 50 PFUs (for mouse serum) of SARS-CoV-2 with the diluted serum samples, and the mixtures were incubated at 37 °C for 1 h. Vero E6 cells in 12-well plates (2 × 10^5^ cells/well) were inoculated with the virus–serum mixtures, and the plates were incubated at 37 °C under 5% CO_2_ for 1 h. After virus absorption, 1.2% agar overlay medium was added, and the plates were incubated at 37 °C under 5% CO_2_ for 2 or 3 days for cell fixation. Plaques were counted after staining with crystal violet mixture.

### IFN-γ enzyme-linked immunospot assay

The ELISpot assay was performed using splenocytes isolated from mice 5 weeks after the first vaccination (2 weeks after the second vaccination). IFN-γ–secreting cells were detected using the mouse IFN-gamma ELISpot kit (XEL485, R&D System). The assays were performed according to the manufacturer’s instructions. Splenocytes (2.5 × 10^5^ cells/well) were seeded on a 96-well polyvinylidene fluoride-backed microplate coated with a monoclonal antibody specific for mouse IFN-γ (890894, R&D Systems). Splenocytes were stimulated with 100 ng/well SARS-CoV-2 spike Glycoprotein-crude (RP30020, GenScript). Tests were conducted with a negative control containing only the medium and a positive control (Cell Stimulation Cocktail; 00-4970-93; eBioScience). Cells were incubated in a 37 °C incubator for 18–24 h. The plate from which cells were removed was washed with a wash buffer (895308, R&D Systems), and a biotinylated monoclonal antibody specific for mouse IFN- **γ** (890895, R&D Systems) was added to each well and incubated for 2 h at room temperature on a rocking platform. After washing, the plates were incubated with streptavidin conjugated to alkaline phosphatase (895358, R&D Systems) for 2 h. The plates were incubated with BCIP/NBT Substrate (895867, R&D Systems) for 1 h at room temperature. Spots were counted using an ImmunoSpot reader.

## Data Availability

The original contributions presented in the study are included in the article/**Supplementary Material**, further inquiries can be directed to the corresponding author/s.

## Supplementary material

Supplementary Figure 1 Characterization of Css_dsg S. **(a, b)** Molecular docking of antigens-antibody 4A8 complexes. The structural model of the antigen-antibody complex was obtained using HADDOCK server. The antigen is shown in green, and the antibody is shown in magenta and yellow, with the heavy and light chains labelled separately. Css_dsg S **(b)** exhibited a higher binding affinity to 4A8 compared to the original Css S **(a)**. **(c)**, Western blot analysis of *in silico*-designed spike expression. Western blot validation of spike protein expression in HEK293T cells transfected with mRNA encoding Ctrl S, Css S, and Css_dsg S. Protein expression was assessed at 24 h after transfection. **(d)**, Relative spike protein expression levels with quantitative values of western blot analysis. The intensity of the bands in (c) was quantified to the expression of Control S using ImageJ software.

Supplementary Figure 2 Humoral immune responses induced by *in silico*-designed mRNA vaccines in BALB/c mice. PRNT assays measuring neutralizing antibody titers in sera from BALB/c mice vaccinated with Css_dsg S, Css S, or Control S against the ancestral, Delta, and BA.5 virus.

Supplementary Figure 3 Survival **(a)** and body weight changes **(b)** and of K18-hACE-2 mice following challenge with the ancestral strain (left) and Delta variant (right). Relative weight was measured as a percentage of initial weight at the indicated days post-infection with the SARS-CoV-2 virus. Body weight and survival were monitored daily for 14 days.

Supplementary Figure 4 Structural modeling of Omi spike protein and antibody binding interactions. **(a, b)** Computational modeling of the designed spike proteins and their interactions with neutralizing antibodies. The trimeric N-terminal domain (NTD) of Omi S **(a)** and Omi_dsg S **(b)** was modeled and docked with the monoclonal antibody 4A8 using AlphaFold2. Structural comparison revealed enhanced binding affinity in Omi_dsg S, demonstrating potential antigenic improvements.

Supplementary Figure 5 Amino acid sequences of the NTD from the control spike (ancestral spike; Control S), consensus-designed spike (Css_dsg S), Omicron-based designed spike (Omi_dsg S), JN.1 spike wild-type (JN.1 _WT), and JN.1 spike_all 12 NTD mutations (JN.1_12muts) were aligned. Stabilizing mutations introduced through *in silico* design are highlighted in red (for Q173 and S256 related mutations) and green (other stabilizing mutations). Dashes indicate deletions. Residue positions correspond to numbering based on the ancestral Control spike sequence.

Supplementary Figure 6 Survival **(a)** and body weight changes **(b)** and of K18-hACE-2 mice following challenge with the ancestral virus. Relative weight was measured as a percentage of initial weight at the indicated days post-infection with the SARS-CoV-2 virus. Body weight and survival were monitored daily for 14 days.

Supplementary Table 1 Properties of seven candidate sequences (D1–D5, DC_Top5 and DC_Tot) assessed by protein stability (energy; free energy of protein conformation from Rosetta energy calculation on PROSS), solubility, RNA 2D stability, and cavity volume.

Supplementary Table 2 Computational comparison of Omi S and in silico designed Omi_dsg S. Energy: free energy of protein structure from Rosetta relax calculation; Solubility: predicted protein solubility; RNA 2D: minimum free energy of RNA secondary structure; and Cavity: internal cavity volume within the protein.

Supplementary Table 3 Properties of seven candidate sequences for Omi_dsg S evaluated by protein stability (Energy: free energy of protein structure from Rosetta relax calculation), solubility, RNA 2D, and cavity volume.

Supplementary Table 4 Mutation sites in Css_dsg S and Omi_dsg S aligned to Control S.

## References

[1] WHO . 14.9 million excess deaths associated with the COVID-19 pandemic in 2020 and 2021. (2022).

[2] Carabelli AM Peacock TP Thorne LG Harvey WT Hughes J de Silva TI . SARS-CoV-2 variant biology: immune escape, transmission and fitness. Nat Rev Microbiol. (2023) 21:162–77. doi: 10.1038/s41579-022-00841-7, PMID: 36653446 PMC9847462

[3] da Costa CHS de Freitas CAB Alves CN Lameira J . Assessment of mutations on RBD in the Spike protein of SARS-CoV-2 Alpha, Delta and Omicron variants. Sci Rep. (2022) 12:8540. doi: 10.1038/s41598-022-12479-9, PMID: 35595778 PMC9121086

[4] Chatterjee S Bhattacharya M Nag S Dhama K Chakraborty C . A detailed overview of SARS-coV-2 omicron: its sub-variants, mutations and pathophysiology, clinical characteristics, immunological landscape, immune escape, and therapies. Viruses. (2023) 15:167. doi: 10.3390/v15010167, PMID: 36680207 PMC9866114

[5] WHO . Initial risk evaluation of JN.1, 19 december 2023. (2023).

[6] J.C. KC Lambrou AS Rose EB Cook PW Batra D Cubenas C . Genomic surveillance for SARS-CoV-2 variants: circulation of omicron XBB and JN.1 lineages — United states, may 2023–September 2024. Atlanta, GA, USA: Centers for Disease Control and Prevention (CDC) (2024). 10.15585/mmwr.mm7342a1 PMC11500842 39446667

[7] Hajizadeh F Khanizadeh S Khodadadi H Mokhayeri Y Ajorloo M Malekshahi A . SARS-COV-2 RBD (Receptor binding domain) mutations and variants (A sectional-analytical study). Microbial Pathogen. (2022) 168:105595. doi: 10.1016/j.micpath.2022.105595, PMID: 35597364 PMC9116045

[8] Harvey WT Carabelli AM Jackson B Gupta RK Thomson EC Harrison EM . SARS-coV-2 variants. spike mutat Immune escape Nat Rev Microbiol. (2021) 19:409–24. doi: 10.1038/s41579-021-00573-0, PMID: 34075212 PMC8167834

[9] Chi X Yan R Zhang J Zhang G Zhang Y Hao M . A neutralizing human antibody binds to the N-terminal domain of the Spike protein of SARS-CoV-2. Science. (2020) 369:650–5. doi: 10.1126/science.abc6952, PMID: 32571838 PMC7319273

[10] Li T Han X Gu C Guo H Zhang H Wang Y . Potent SARS-CoV-2 neutralizing antibodies with protective efficacy against newly emerged mutational variants. Nat Commun. (2021) 12:6304. doi: 10.1038/s41467-021-26539-7, PMID: 34728625 PMC8563728

[11] Xie X Liu Y Liu J Zhang X Zou J Fontes-Garfias CR . Neutralization of SARS-CoV-2 spike 69/70 deletion, E484K and N501Y variants by BNT162b2 vaccine-elicited sera. Nat Med. (2021) 27:620–1. doi: 10.1038/s41591-021-01270-4, PMID: 33558724

[12] Polack FP Thomas SJ Kitchin N Absalon J Gurtman A Lockhart S . Safety and efficacy of the BNT162b2 mRNA covid-19 vaccine. New Engl J Med. (2020) 383:2603–15. doi: 10.1056/NEJMoa2034577, PMID: 33301246 PMC7745181

[13] Sahin U Muik A Vogler I Derhovanessian E Kranz LM Vormehr M . BNT162b2 vaccine induces neutralizing antibodies and poly-specific T cells in humans. Nature. (2021) 595:572–7. doi: 10.1038/s41586-021-03653-6, PMID: 34044428

[14] Kuodi P Gorelik Y Zayyad H Wertheim O Wiegler KB Abu Jabal K . Association between BNT162b2 vaccination and reported incidence of post-COVID-19 symptoms: cross-sectional study 2020-21, Israel. NPJ Vaccines. (2022) 7:101. doi: 10.1038/s41541-022-00526-5, PMID: 36028498 PMC9411827

[15] Baden LR El Sahly HM Essink B Kotloff K Frey S Novak R . Efficacy and safety of the mRNA-1273 SARS-coV-2 vaccine. New Engl J Med. (2021) 384:403–16. doi: 10.1056/NEJMoa2035389, PMID: 33378609 PMC7787219

[16] El Sahly HM Baden LR Essink B Doblecki-Lewis S Martin JM Anderson EJ . Efficacy of the mRNA-1273 SARS-CoV-2 Vaccine at Completion of Blinded Phase. New Engl J Med. (2021) 385:1774–85. doi: 10.1056/NEJMoa2113017, PMID: 34551225 PMC8482810

[17] Chemaitelly H Yassine HM Benslimane FM Al Khatib HA Tang P Hasan MR . mRNA-1273 COVID-19 vaccine effectiveness against the B.1.1.7 and B.1.351 variants and severe COVID-19 disease in Qatar. Nat Med. (2021) 27:1614–21. doi: 10.1038/s41591-021-01446-y, PMID: 34244681

[18] Weidenbacher PA Friedland N Sanyal M Morris MK Do J Hanson C . Decreased efficacy of a COVID-19 vaccine due to mutations present in early SARS-CoV-2 variants of concern. bioRxiv. (2023). doi: 10.1101/2023.06.27.546764, PMID: 37425802 PMC10326996

[19] Tegally H Wilkinson E Giovanetti M Iranzadeh A Fonseca V Giandhari J . Detection of a SARS-CoV-2 variant of concern in South Africa. Nature. (2021) 592:438–43. doi: 10.1038/s41586-021-03402-9, PMID: 33690265

[20] Lasrado N Rowe M McMahan K Hachmann NP Miller J Jacob-Dolan C . SARS-CoV-2 XBB.1.5 mRNA booster vaccination elicits limited mucosal immunity. Sci Trans Med. (2024) 16:eadp8920. doi: 10.1126/scitranslmed.adp8920, PMID: 39441905 PMC11542980

[21] Kumari M Su SC Liang KH Lin HT Lu YF Chen KC . Bivalent mRNA vaccine effectiveness against SARS-CoV-2 variants of concern. J Biomed Sci. (2023) 30:46. doi: 10.1186/s12929-023-00936-0, PMID: 37380988 PMC10304269

[22] Yun JS Kim AR Kim SM Shin E Ha SJ Kim D . In silico analysis for the development of multi-epitope vaccines against Mycobacterium tuberculosis. Front Immunol. (2024) 15:1474346. doi: 10.3389/fimmu.2024.1474346, PMID: 39624097 PMC11609213

[23] Al Tbeishat H . Novel In Silico mRNA vaccine design exploiting proteins of M. tuberculosis that modulates host immune responses by inducing epigenetic modifications. Sci Rep. (2022) 12:4645. doi: 10.1038/s41598-022-08506-4, PMID: 35301360 PMC8929471

[24] Shi J Zhu Y Yin Z He Y Li Y Haimiti G . In silico designed novel multi-epitope mRNA vaccines against Brucella by targeting extracellular protein BtuB and LptD. Sci Rep. (2024) 14:7278. doi: 10.1038/s41598-024-57793-6, PMID: 38538674 PMC10973489

[25] Khan K Yaqub T Shabbir MZ Aslam A Mukhtar N Fazal S . In-silico vaccine matching and its validation through in-vivo immune protection analysis for imported and indigenous vaccines against recent field isolate of avian influenza H9N2. Vet Vaccine. (2023) 2:100029. doi: 10.1016/j.vetvac.2023.100029

[26] Zhang J Xiao T Cai Y Chen B . Structure of SARS-coV-2. spike protein Curr Opin Virol. (2021) 50:173–82. doi: 10.1016/j.coviro.2021.08.010, PMID: 34534731 PMC8423807

[27] McCallum M De Marco A Lempp FA Tortorici MA Pinto D Walls AC . N-terminal domain antigenic mapping reveals a site of vulnerability for SARS-CoV-2. Cell. (2021) 184:2332–2347.e16. doi: 10.1016/j.cell.2021.03.028, PMID: 33761326 PMC7962585

[28] Cerutti G Guo Y Zhou T Gorman J Lee M Rapp M . Potent SARS-CoV-2 neutralizing antibodies directed against. spike N-terminal domain targ single supers Cell Host Microbe. (2021) 29:819–833.e7. doi: 10.1016/j.chom.2021.03.005, PMID: 33789084 PMC7953435

[29] Jumper J Evans R Pritzel A Green T Figurnov M Ronneberger O . Highly accurate protein structure prediction with AlphaFold. Nature. (2021) 596:583–9. doi: 10.1038/s41586-021-03819-2, PMID: 34265844 PMC8371605

[30] Lorenz R Bernhart SH Höner Zu Siederdissen C Tafer H Flamm C Stadler PF . Assessing and enhancing foldability in designed proteins. Protein Sci. (2022) 31:e4400. doi: 10.1002/pro.4400, PMID: 36040259 PMC9375437

[31] Khersonsky O Lipsh R Avizemer Z Ashani Y Goldsmith M Leader H . Automated design of efficient and functionally diverse enzyme repertoires. Mol Cell. (2018) 72:178–186.e5. doi: 10.1016/j.molcel.2018.08.033, PMID: 30270109 PMC6193528

[32] Delano WL . The pyMOL molecular graphics system. (2002).

[33] Kuriata A Iglesias V Pujols J Kurcinski M Kmiecik S Ventura S . Aggrescan3D (A3D) 2.0: prediction and engineering of protein solubility. Nucleic Acids Res. (2019) 47:W300–w307. doi: 10.1093/nar/gkz321, PMID: 31049593 PMC6602499

[34] Tian W Chen C Lei X Zhao J Liang J . CASTp 3.0: computed atlas of surface topography of proteins. Nucleic Acids Res. (2018) 46:W363–w367. doi: 10.1093/nar/gky473, PMID: 29860391 PMC6031066

[35] Lorenz R Bernhart SH Höner Zu Siederdissen C Tafer H Flamm C Stadler PF . ViennaRNA package 2.0. Algorith Mol Biol. (2011) 6:26. doi: 10.1186/1748-7188-6-26, PMID: 22115189 PMC3319429

[36] Zhang H Zhang L Lin A Xu C Li Z Liu K . Algorithm for optimized mRNA design improves stability and immunogenicity. Nature. (2023) 621:396–403. doi: 10.1038/s41586-023-06127-z, PMID: 37130545 PMC10499610

[37] Evans R O’Neill M Pritzel A Antropova N Senior A Green T . Protein complex prediction with AlphaFold-Multimer. bioRxiv. (2022), 2021.10.04.463034. doi: 10.1101/2021.10.04.463034

[38] Honorato RV Trellet ME Jiménez-García B Schaarschmidt JJ Giulini M Reys V . The HADDOCK2.4 web server for integrative modeling of biomolecular complexes. Nat Protoc. (2024) 19:3219–41. doi: 10.1038/s41596-024-01011-0, PMID: 38886530

[39] Xue LC Rodrigues JP Kastritis PL Bonvin AM Vangone A . PRODIGY: a web server for predicting the binding affinity of protein-protein complexes. Bioinformatics. (2016) 32:3676–8. doi: 10.1093/bioinformatics/btw514, PMID: 27503228

[40] Roney JP Ovchinnikov S . State-of-the-art estimation of protein model accuracy using alphafold. Phys Rev Lett. (2022) 129:238101. doi: 10.1103/PhysRevLett.129.238101, PMID: 36563190 PMC12178128

[41] Williams CJ Headd JJ Moriarty NW Prisant MG Videau LL Deis LN . MolProbity: More and better reference data for improved all-atom structure validation. Protein Sci. (2018) 27:293–315. doi: 10.1002/pro.3330, PMID: 29067766 PMC5734394

