# Supplementary figures and images for: Structure-guided design of a bivalent SARS-CoV-2 mRNA vaccine with NTD stabilizing mutations enhances broad immunity
Source: Front Immunol. 2026 Jan 22;16:1718740. doi: 10.3389/fimmu.2025.1718740 (PMC12872526; doi:10.3389/fimmu.2025.1718740)

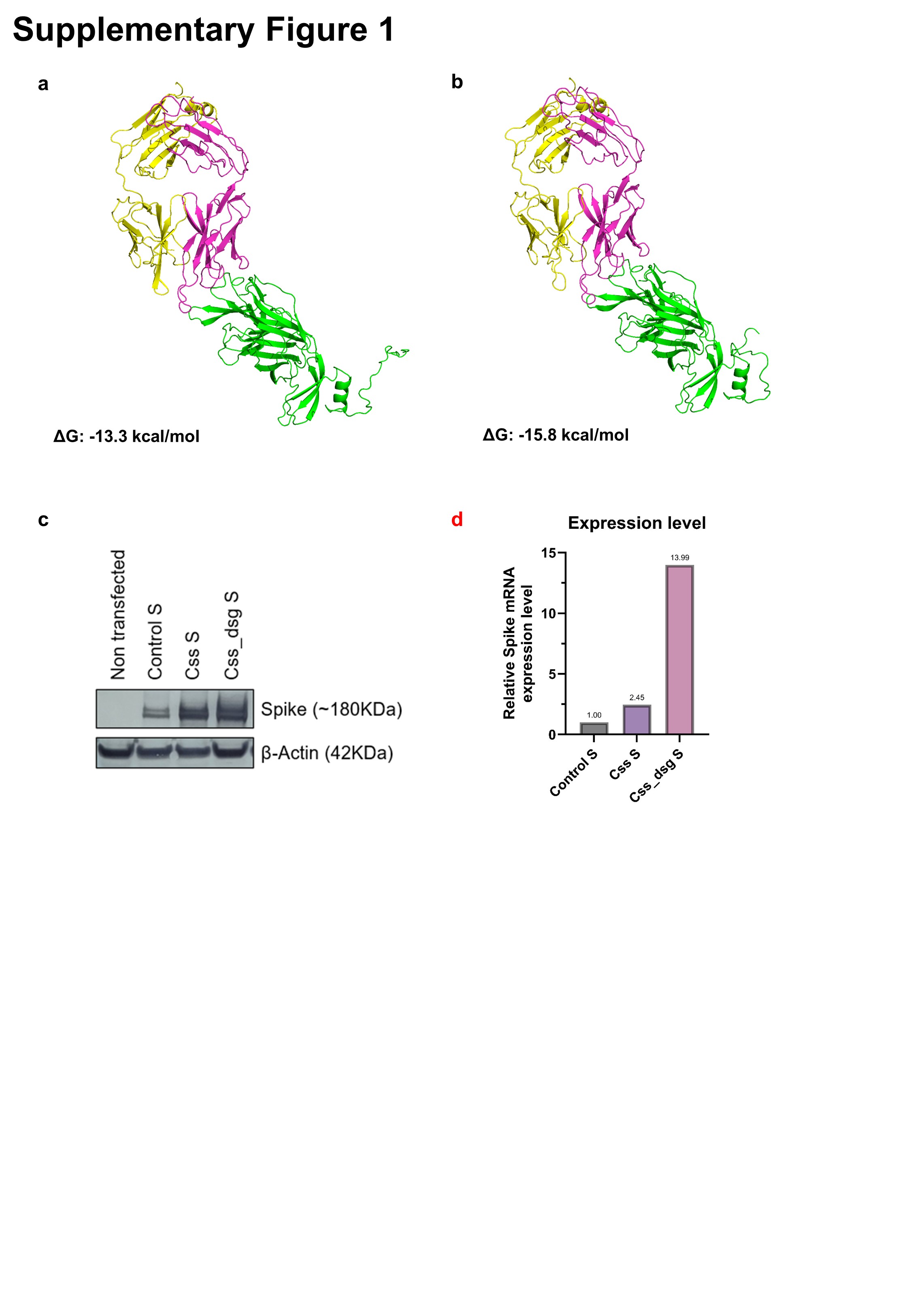

Supplement: Supplementary Figure 1 — Characterization of Css_dsg S. (a, b) Molecular docking of antigens-antibody 4A8 complexes. The structural model of the antigen-antibody complex was obtained using HADDOCK server. The antigen is shown in green, and the antibody is shown in magenta and yellow, with the heavy and light chains labelled separately. Css_dsg S (b) exhibited a higher binding affinity to 4A8 compared to the original Css S (a). (c), Western blot analysis of in silico-designed spike expression. Western blot validation of spike protein expression in HEK293T cells transfected with mRNA encoding Ctrl S, Css S, and Css_dsg S. Protein expression was assessed at 24 h after transfection. (d), Relative spike protein expression levels with quantitative values of western blot analysis. The intensity of the bands in (c) was quantified to the expression of Control S using ImageJ software. [file Image1.jpeg]

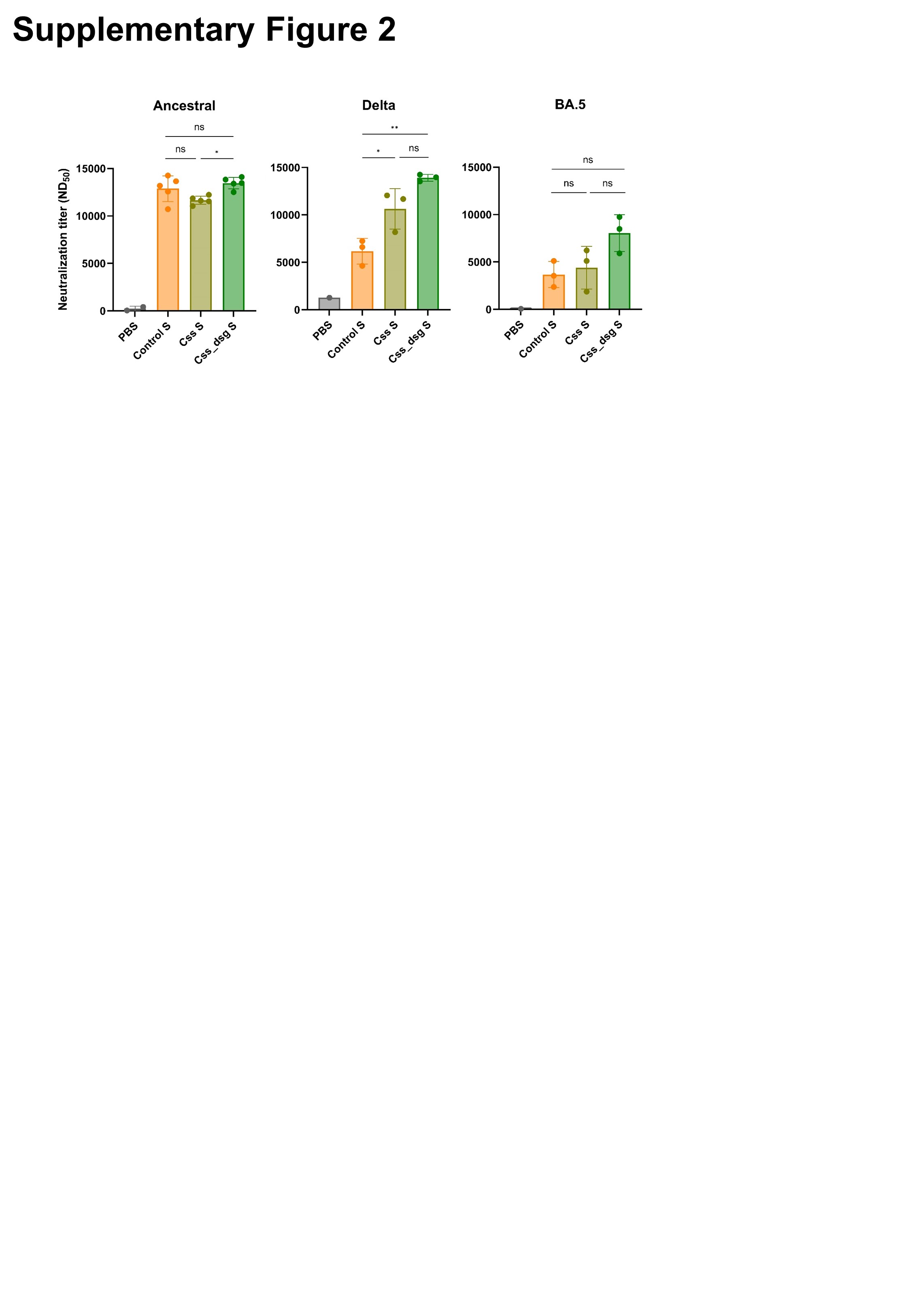

Supplement: Supplementary Figure 2 — Humoral immune responses induced by in silico-designed mRNA vaccines in BALB/c mice. PRNT assays measuring neutralizing antibody titers in sera from BALB/c mice vaccinated with Css_dsg S, Css S, or Control S against the ancestral, Delta, and BA.5 virus. [file Image2.jpeg]

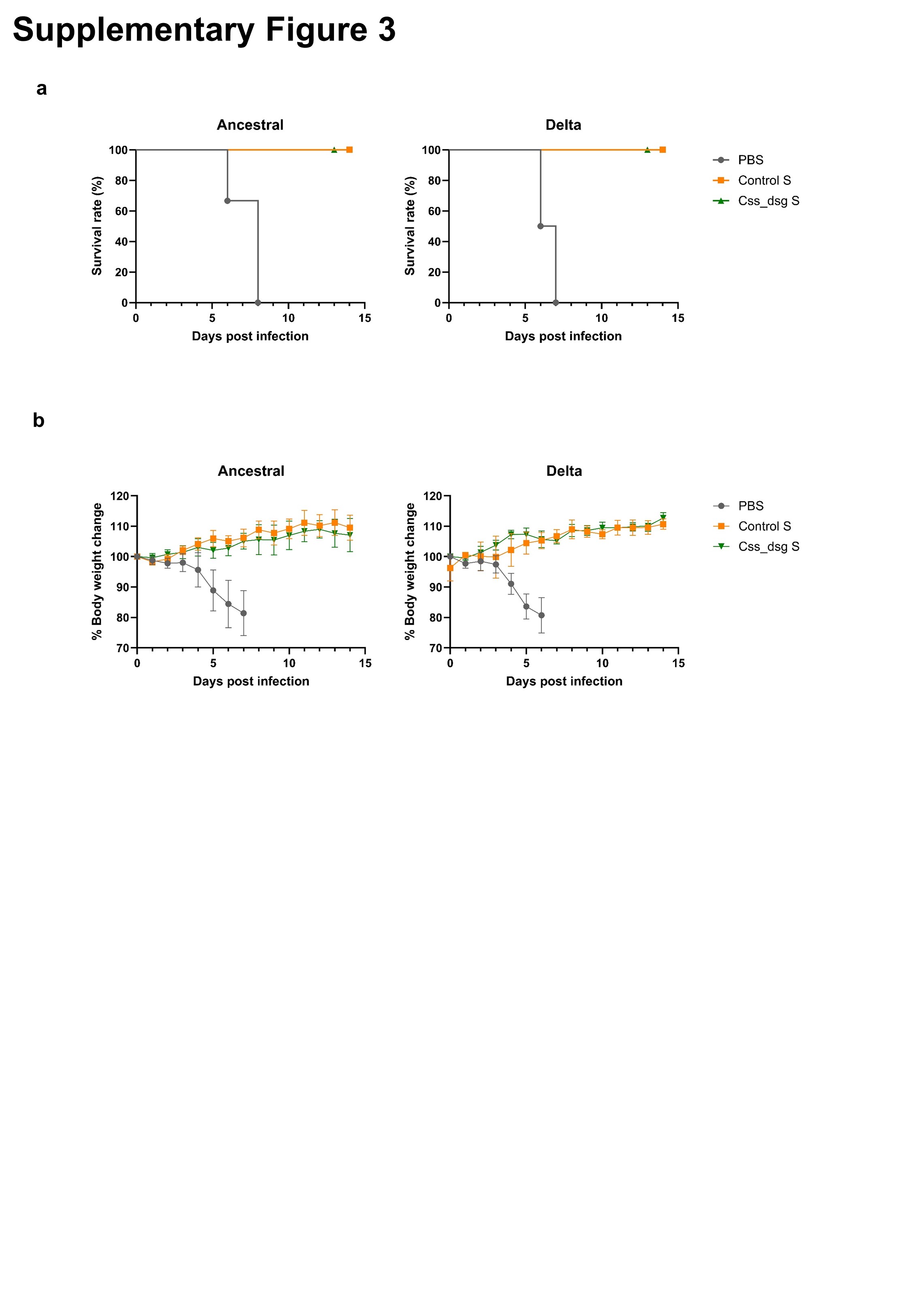

Supplement: Supplementary Figure 3 — Survival (a) and body weight changes (b) and of K18-hACE-2 mice following challenge with the ancestral strain (left) and Delta variant (right). Relative weight was measured as a percentage of initial weight at the indicated days post-infection with the SARS-CoV-2 virus. Body weight and survival were monitored daily for 14 days. [file Image3.jpeg]

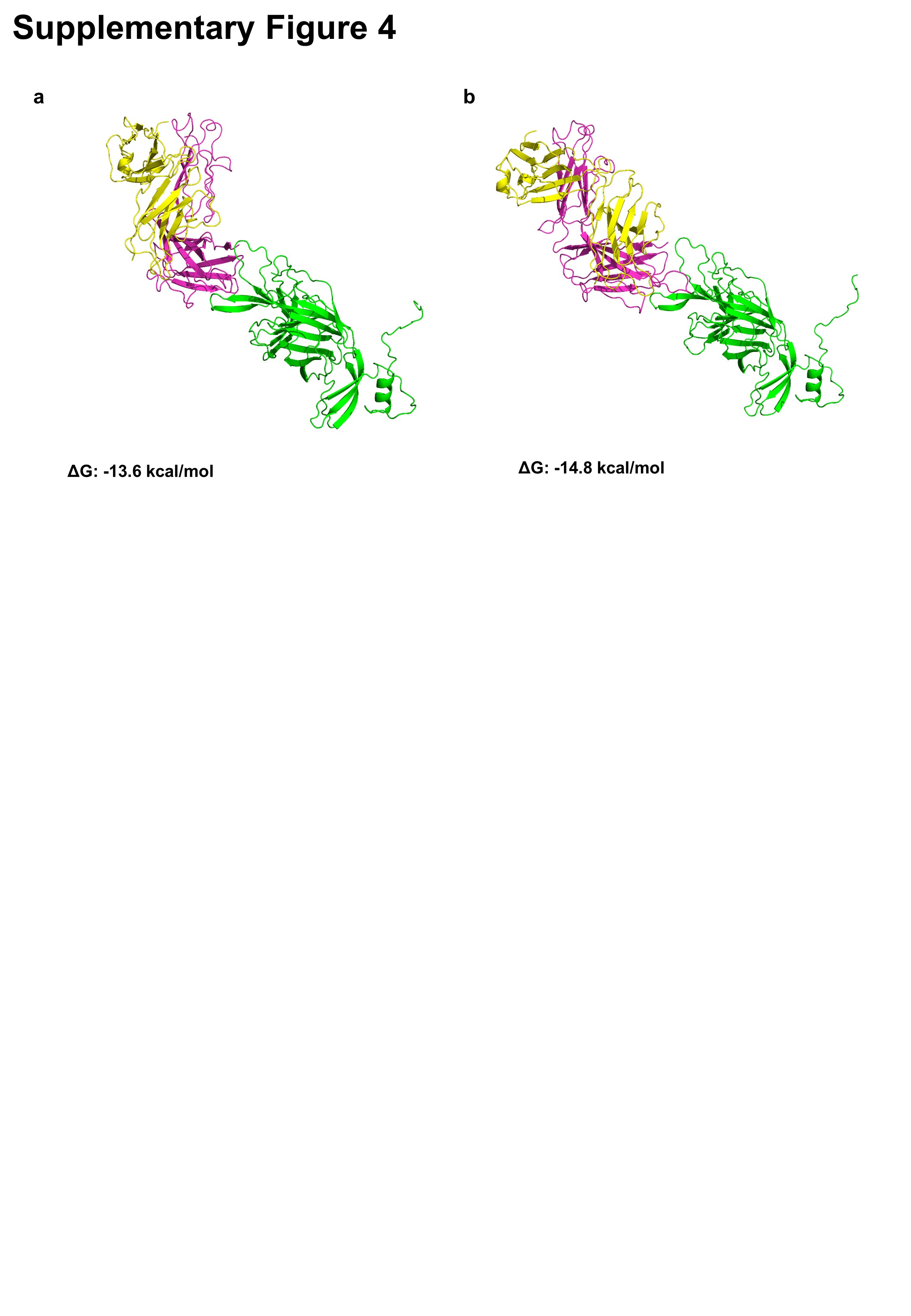

Supplement: Supplementary Figure 4 — Structural modeling of Omi spike protein and antibody binding interactions. (a, b) Computational modeling of the designed spike proteins and their interactions with neutralizing antibodies. The trimeric N-terminal domain (NTD) of Omi S (a) and Omi_dsg S (b) was modeled and docked with the monoclonal antibody 4A8 using AlphaFold2. Structural comparison revealed enhanced binding affinity in Omi_dsg S, demonstrating potential antigenic improvements. [file Image4.jpeg]

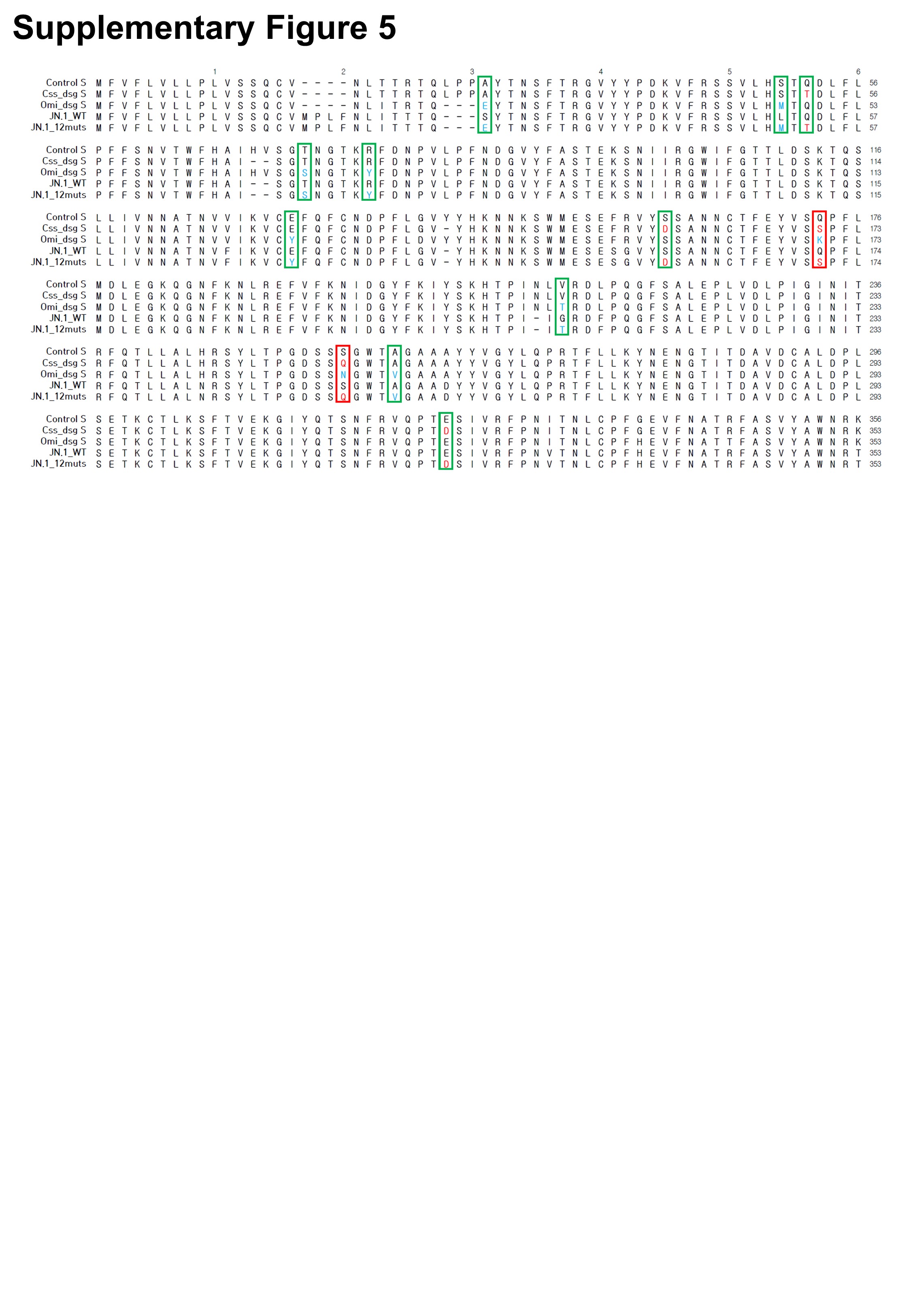

Supplement: Supplementary Figure 5 — Amino acid sequences of the NTD from the control spike (ancestral spike; Control S), consensus-designed spike (Css_dsg S), Omicron-based designed spike (Omi_dsg S), JN.1 spike wild-type (JN.1 _WT), and JN.1 spike_all 12 NTD mutations (JN.1_12muts) were aligned. Stabilizing mutations introduced through in silico design are highlighted in red (for Q173 and S256 related mutations) and green (other stabilizing mutations). Dashes indicate deletions. Residue positions correspond to numbering based on the ancestral Control spike sequence. [file Image5.jpeg]

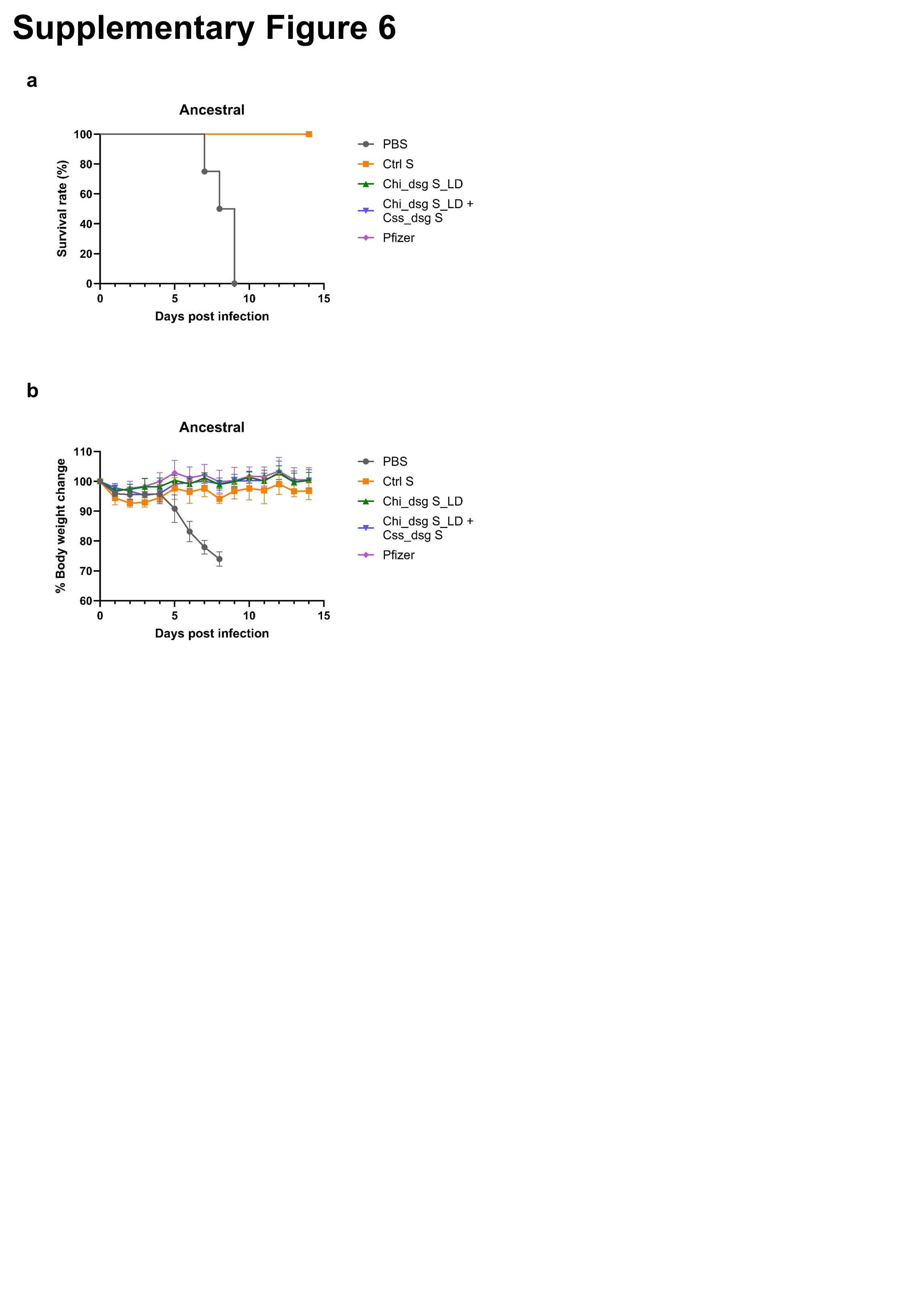

Supplement: Supplementary Figure 6 — Survival (a) and body weight changes (b) and of K18-hACE-2 mice following challenge with the ancestral virus. Relative weight was measured as a percentage of initial weight at the indicated days post-infection with the SARS-CoV-2 virus. Body weight and survival were monitored daily for 14 days. [file Image6.jpeg]

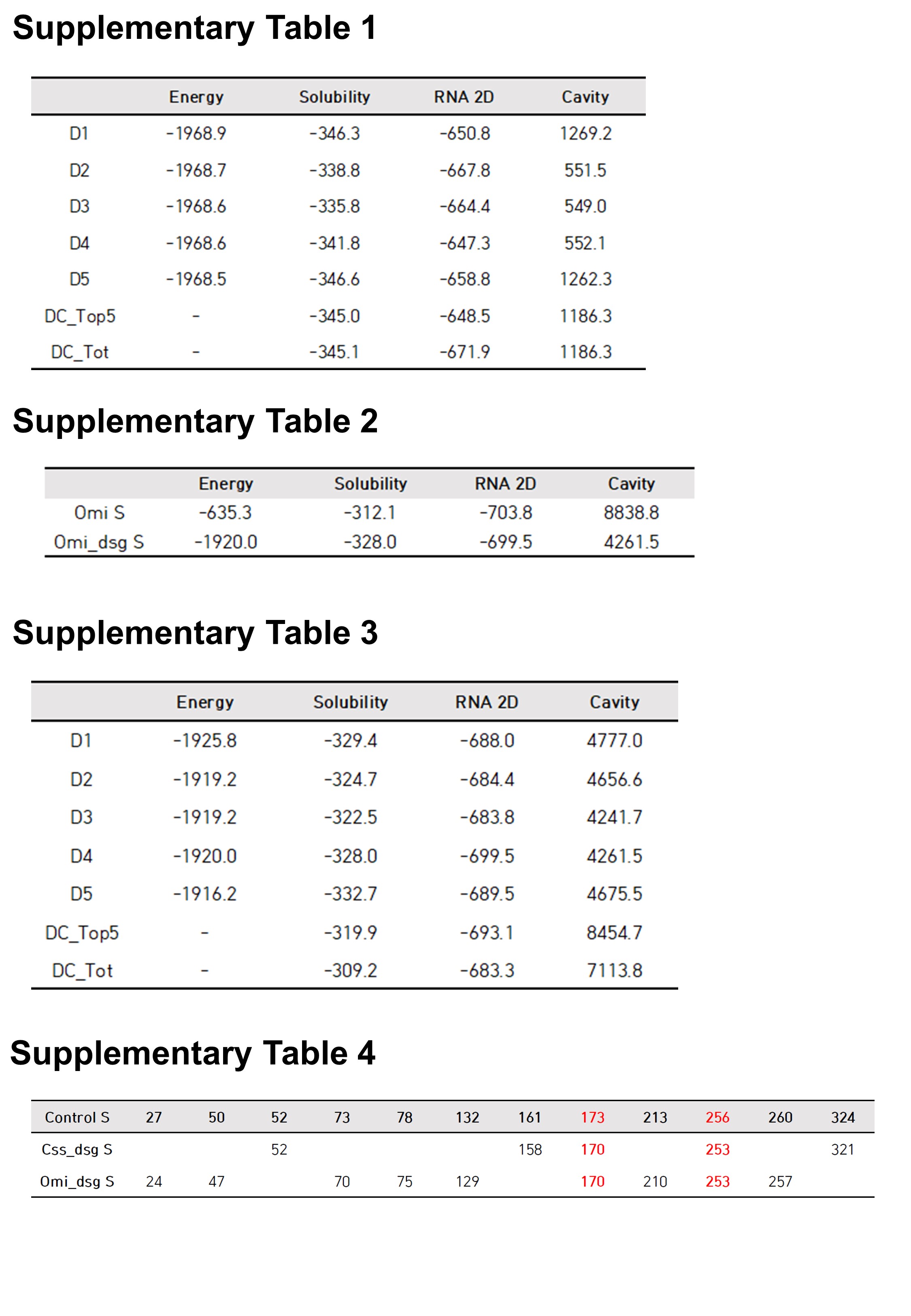

Supplement: Supplementary Table 1 — Properties of seven candidate sequences (D1–D5, DC_Top5 and DC_Tot) assessed by protein stability (energy; free energy of protein conformation from Rosetta energy calculation on PROSS), solubility, RNA 2D stability, and cavity volume. [file SupplementaryFile1.jpeg]
